# Selective Response to Bacterial Infection by Regulating Siglec-E Expression

**DOI:** 10.1016/j.isci.2020.101473

**Published:** 2020-08-20

**Authors:** Yin Wu, Darong Yang, Runhua Liu, Lizhong Wang, Guo-Yun Chen

**Affiliations:** 1 Children's Foundation Research Institute at Le Bonheur Children's Hospital, Department of Pediatrics, University of Tennessee Health Science Center, Memphis, TN 38103, USA; 2 Department of Genetics, University of Alabama at Birmingham, Birmingham, AL, USA; 3 Comprehensive Cancer Center, University of Alabama at Birmingham, Birmingham, AL, USA

**Keywords:** Genetics, Molecular Biology, Microbiology

## Abstract

Interactions between microbes and hosts can be a benign, deleterious, or even fatal, resulting in death of the host, the microbe, or both. Sialic acid-binding immunoglobulin-like lectins (Siglecs) suppress infection responses to sialylated pathogens. However, most pathogens are nonsialylated. Here we determined Siglecs respond to nonsialylated Gram-negative bacteria (*Escherichia coli 25922* and *DH5α*) and Gram-positive bacteria (*Staphylococcus aureus* and *Listeria monocytogenes*). We found that *Siglece*^*−/−*^ mice had higher mortality than wild-type mice following Gram-negative but not Gram-positive bacterial infection. Better survival in wild-type mice depended on more efficient clearance of Gram-negative than Gram-positive bacteria. Gram-negative bacteria upregulated Siglec-E, thus increasing reactive oxygen species (ROS); Tyr432 in the ITIM domain of Siglec-E was required to increase ROS. Moreover, Gram-negative bacteria upregulated Siglec-E via TLR4/MyD88/JNK/NF-κB/AP-1, whereas Gram-positive bacteria downregulated Siglec-E via TLR2/RANKL/TRAF6/Syk. Thus, our study describes a fundamentally new role for Siglec-E during infection.

## Highlights

• Siglec-E controls bacterial survival by regulating ROS generation
• Gram-negative bacteria upregulated Siglec-E via TLR4/MyD88/JNK/NF-κB/AP-1
• Gram-positive bacteria downregulated Siglec-E via TLR2/RANKL/TRAF6/Syk

## Graphical Abstract

**Figure:**
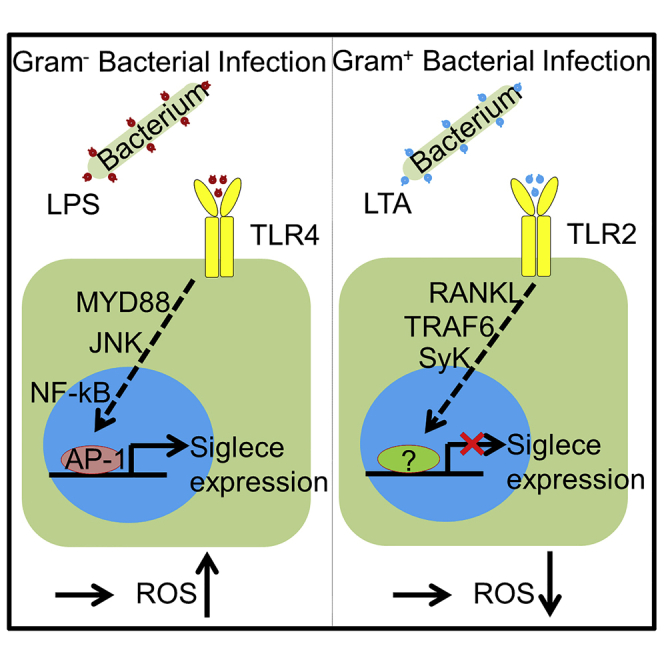

## Introduction

Interactions between host molecules and bacterial antigens are dynamic and can be benign, deleterious, or even fatal, resulting in the death of the host, microbe, or both (Merrell and Falkow, 2004; Ottemann and Kenney, 2019; Medzhitov, 2007; Bhavsar et al., 2007; Casadevall and Pirofski, 2000). Many microbial pathogens avoid host recognition or dampen subsequent immune activation through interactions with host responses, but some pathogens benefit from stimulating inflammatory responses (Vimr and Lichtensteiger, 2002). Sialic acids are a family of nine-carbon sugars, and N-Glycolylneuraminic acid (Neu5Gc) and N-acetylneuraminic acid (Neu5Ac) are major sialic acids (Chen et al., 2014b). In mammalian cells, sialic acid is usually the terminal sugar residue on the oligosaccharide chains of cell-surface glycopeptides or glycolipids, where it functions in recognition and anti-recognition in regulation of cell-cell interactions (Chen et al., 2014b). Although some oropharyngeal pathogens express sialic acid units on their surfaces, mimicking the sialyl-rich mucin layer coating host epithelial cells to masquerade as “self” while eluding host immune mechanisms, most microbes do not express sialic acid on their surface (Vimr and Lichtensteiger, 2002). How hosts respond to nonsialylated microbial pathogens is poorly understood.

The host's response to a pathogen involves both the innate and adaptive immune systems, with Toll-like receptors (TLRs) playing an important role. TLRs recognize conserved structures in pathogens and have revealed how the body senses pathogen invasion, triggers innate immune responses, and primes antigen-specific adaptive immunity (Akira and Takeda, 2004; Liew et al., 2005; Trinchieri and Sher, 2007; Barton and Kagan, 2009; Kawai and Akira, 2010, 2011; Mills, 2011; Kondo et al., 2012). TLRs are divided into two groups based on their cellular localization and pathogen-associated molecular pattern (PAMP) ligands. One group, including TLR1, TLR2, TLR4, TLR5, TLR6, and TLR11, is expressed on cell surfaces and recognizes microbial membrane components such as lipids, lipoproteins, and proteins. The other group, including TLR3, TLR7, TLR8, and TLR9, is expressed exclusively in intracellular vesicles such as the endoplasmic reticulum (ER), endosomes, lysosomes, and endolysosomes, where they recognize microbial nucleic acids (Akira and Takeda, 2004; Liew et al., 2005; Trinchieri and Sher, 2007; Barton and Kagan, 2009; Kawai and Akira, 2010, 2011; Mills, 2011; Kondo et al., 2012). TLR4 responds to bacterial lipopolysaccharide (LPS), an outer membrane component of Gram-negative bacteria that can cause septic shock. TLR2 contributes to the recognition of a wide range of PAMPs derived from bacteria, fungi, parasites, and viruses. These PAMPs include lipopeptides and peptidoglycan from bacteria and lipoteichoic acid (LTA) from Gram-positive bacteria (Akira and Takeda, 2004; Liew et al., 2005; Trinchieri and Sher, 2007; Barton and Kagan, 2009; Kawai and Akira, 2010, 2011; Mills, 2011; Kondo et al., 2012). In this article, we focus on the mechanisms involved in the pathogenesis and host responses to two pathogens: the Gram-negative bacteria *Escherichia coli 25922* and *DH5α* and the Gram-positive bacteria *Staphylococcus aureus* and *Listeria monocytogenes*.

Siglecs are membrane-bound lectins comprising the sialic acid-binding immunoglobulin superfamily, and each Siglec has a distinct cellular distribution and glycan specificities (Crocker et al., 2007). Siglecs predominantly bind to sialic acids on cell surface proteins (Crocker, 2002) and participate in the internalization of sialic acid-expressing pathogens (Yu et al., 2014; Tateno et al., 2007; Jones et al., 2003), self-tolerance (Bokers et al., 2014), and endotoxin tolerance (Wu et al., 2016a). Previously, we found an interaction between CD24 and Siglec-G/10 selectively suppresses the inflammatory response to damage-associated molecular patterns (DAMPs) in tissue injury and is a key regulator of polybacterial sepsis. This interaction requires sialylation of CD24 (Chen et al., 2009, 2011). Moreover, the Siglec-G/CD24 axis controls the severity of graft versus host disease (GVHD), and enhancing this interaction may mitigate GVHD (Toubai et al., 2014, 2017). The CD24/siglec-10 signaling pathway protects cancer cells from the immune system, indicating a potential target for cancer immunotherapy (Barkal et al., 2019). The broad spectrum of interaction between Siglecs and TLR further indicates that Siglecs may be the central regulator of the innate immune response (Chen et al., 2014a). Siglec-G regulates inflammation in response to RNA virus infection (Chen et al., 2013), whereas Siglec-9 negatively regulates the innate immune response to sialylated bacterial infection (Carlin et al., 2009). Siglec-E (the human homolog of Siglec-9 in mice) was reported to negatively regulate the inflammatory response (Wu et al., 2016b; Yousif, 2014; McMillan et al., 2013, 2014; Boyd et al., 2009). Siglec-E can also repress the immune response by direct binding to heavily sialylated *Group B streptococcus* via α2-3-linked sialyllactosamine capsular polysaccharide (Chang et al., 2014; Saito et al., 2016; Chang and Nizet, 2014). However, the role of Siglec-E in host defense against nonsialylated microbial pathogens and the signaling pathway involved remain unclear.

Although neutrophils have historically been characterized as first responder cells and are vital for host survival in bacterial infection, the role of neutrophils in fighting bacterial infections remains a critical issue in human pathologies. When microbes penetrate the epithelial barrier, neutrophils are rapidly recruited and upon contact engulf the bacteria into a vacuole (Flannagan et al., 2009; Urban et al., 2006). Neutrophils produce several potent antimicrobial molecules, like reactive oxygen species (ROS), to kill engulfed bacteria (Nguyen et al., 2017; Dahlgren et al., 2019). ROS are essential for host defense and the innate immune response against bacterial infections. Neutrophils generate high levels of ROS using a superoxide-generating NADPH oxidase complex. NOX2, a membrane-bound subunit of the NADPH oxidase complex, is a large protein complex composed of the transmembrane proteins gp91^phox^ and gp22^phox^, as well as three cytosolic components (p40^phox^, p47^phox^, and p67^phox^). NOX2 activation recruits cytosolic subunits to the membrane and mediates sustained ROS production (Brandes et al., 2014; Bedard and Krause, 2007). Although many regulators of ROS production in phagocytes have been described, our knowledge about its precise control is still limited. Here we show Siglec-E controls bacterial survival by regulating ROS generation by neutrophils during bacterial infection.

## Results

### *Siglece*^*−/−*^ Mice Are less Resistant to *E. coli 25922* and *DH5α* but Not *S. aureus* and *L. monocytogenes* Infection Than *Siglece*^*+/+*^ Mice

**Figure 1:**
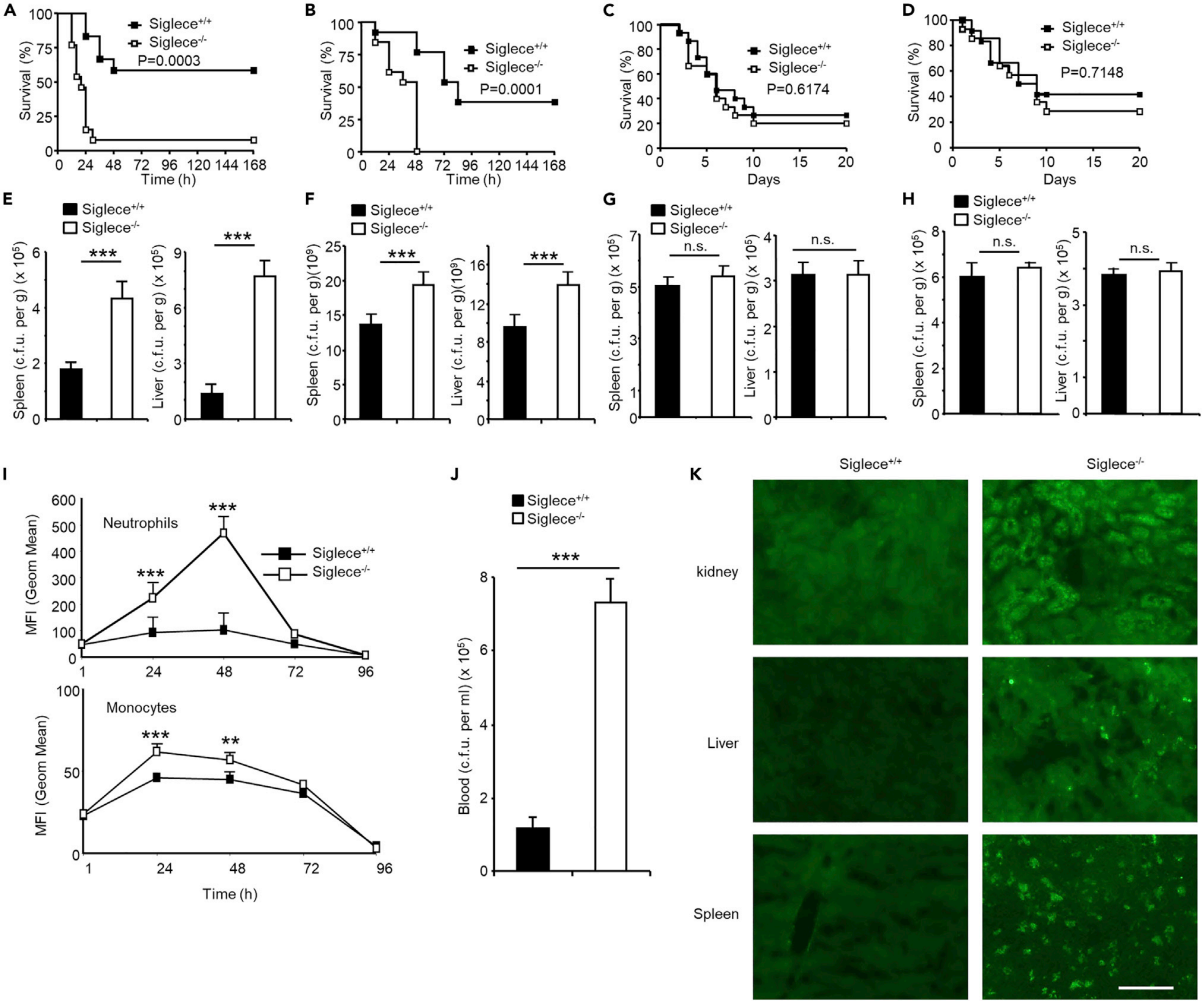
Siglec-E Improves Bacterial Clearance and Survival in Gram-Negative but Not Gram-Positive Bacterial Infection (A–D) Kaplan-Meier curve for Siglece^−/−^ and wild-type littermates after i.p. injection with *E. coli 25922* (n = 8) (A), *DH5α* (n = 8) (B), *S. aureus* (n = 8) (C) and *L. monocytogenes* (n = 8) (D). Log rank (Mantel-Cox) test. Experiments in this figure were reproduced three (A and B) and two (C and D) times. (E–H) Bacterial c.f.u. 16 h after i.p. injection with *E. coli 25922* (E), *DH5α* (F), *S. aureus* (G), and *L. monocytogenes* (H) in liver and spleen (n = 4 each group). (I) Bacterial loads in neutrophils (CD111b^+^Gr-1^+^) and monocytes (CD111b^+^Gr-1^-^) after i.p. infection (2 × 10^5^ c.f.u) with *E. coli 25922GFP* at the indicated time points (n = 4 each group). (J) Bacterial loads in blood after i.v. injection with *E. coli 25922GFP* (n = 5). (K) Liver, kidney, and spleen were collected 16 h post infection (i.v. injection with *E. coli 25922GFP*), and frozen sections were made (n = 3, repeated once). Scale Bar, 50 uM. (E–J) Data are presented as the mean ± SEM from two (G–J) or three (E and F) independent experiments, Student's t test. ∗∗p < 0.01, ∗∗∗p < 0.001, n.s., not significant.

Recent studies indicated Siglec-E represses the immune response by direct binding to heavily sialylated *Group B streptococcus* via α2-3-linked sialyllactosamine capsular polysaccharide (Chang et al., 2014; Saito et al., 2016; Chang and Nizet, 2014). Most pathogens do not contain α2-3 or α2-6 linked sialylation, but some pathogens bear capsules that are polymers of α2-8-linked polysialic acid (PSA) (Devi et al., 1991). To characterize the role of Siglec-E during microbial infections with nonsialylated bacteria, we carried out experiments in Siglec-E deficient mice (as shown in Figures S1A–S1C, the mice were further characterized) (Wu et al., 2016b; Chen et al., 2014a). Siglec-E-deficient and wild-type littermates were infected intraperitoneally (i.p.) with Gram-negative *E. coli 25922* or *DH5α* or Gram-positive *S. aureus* or *L. monocytogenes*. These bacteria are nonsialylated as revealed by staining with *Sambucus nigra* lectin (SNA) and *Maackia amurensis* lectin (MAA) (Figure S2) and showed no binding activity to soluble Siglec-E IgG Fc fusion protein (Figure S3). We found wild-type mice were more likely to survive than *Siglece*^*−/−*^ mice after infection with Gram-negative bacteria, but this advantage was not observed when mice were infected with Gram-positive bacteria (Figure 1A–1D) or treated with LPS (Figure S4).

To dissect the mechanism responsible for the survival disadvantage of *Siglece*^*−/−*^ mice, we determined bacterial burdens in systemic organs 16 h post infection. Notably, the liver and spleen of *Siglece*^*−/−*^ mice contained significantly more bacteria than those of wild-type littermates after infection with *E. coli 25922* and *DH5α* but not with *S. aureus* and *L. monocytogenes* (Figure 1E–1H). Accordingly, *Siglece*^*−/−*^ mice produced more IL-6 and TNF-α than wild-type littermates after infection with *E. coli 25922* or *DH5α* but not with *S. aureus* or *L. monocytogenes* (Figure S5). Pathogen load was also increased at various times after infection with *E. coli 25922GFP* as assessed by measuring the mean fluorescence intensity (MFI) of neutrophils and monocytes (Figure 1I).

To examine the role of Siglec-E in an alternative infection route, we infected *Siglece*^*−/−*^ and wild-type littermates with *E. coli 25922GFP* intravenously (i.v.). Similar to mice infected i.p. (Figures 1E and 1F), *Siglece*^*−/−*^ mice infected i.v. showed significantly higher bacterial burden in the blood, liver, kidney, and spleen than wild-type littermates 16 h post infection, indicating that wild-type littermates cleared *E. coli 25922GFP* more efficiently than *Siglece*^*−/−*^ mice (Figures 1J and 1K).

Nine Siglecs have been identified in mouse; among them, Siglec-E and Siglec-F reportedly mediate uptake of sialylated bacteria (Tateno et al., 2007). Next, we tested the role of Siglec-F in bacterial infection, as it reportedly mediates uptake of sialylated bacteria (Tateno et al., 2007). We found no difference in survival, cytokine production, and bacterial clearance between Siglec-F knockout and wild-type mice with *E. coli 25922* infection (i.p.) (Figure S6), suggesting Siglec-F has no effect on bacterial clearance. These results demonstrate a critical role for Siglec-E but not for Siglec-F in regulating the clearance of bacterial pathogens such as Gram-negative *E. coli 25922* and *DH5α* but not Gram-positive *S. aureus* and *L. monocytogenes*.

### Siglec-E Is Required for Bacterial Clearance but Not Bacterial Uptake

**Figure 2:**
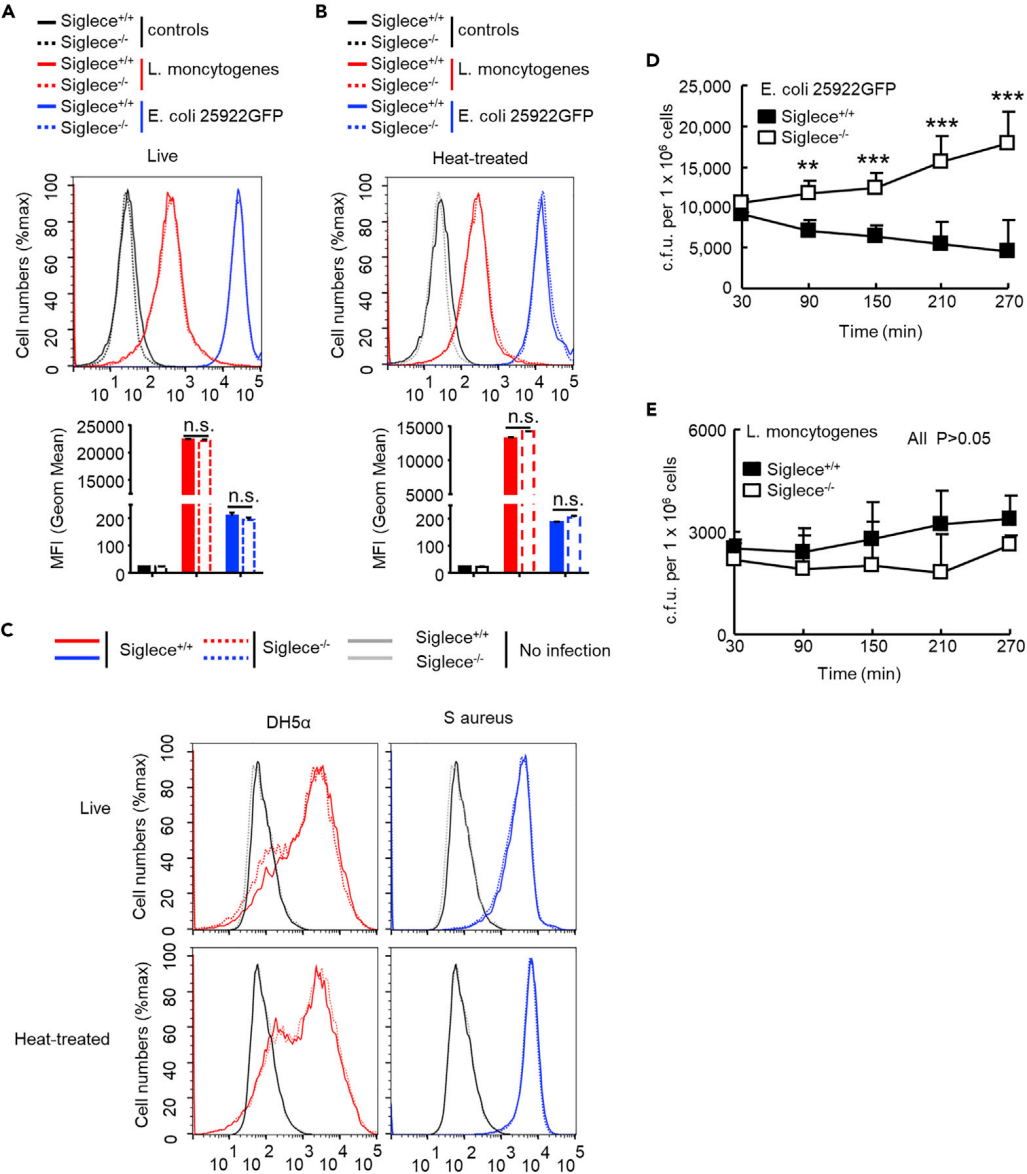
Siglec-E is Required for Efficient Bacterial Clearance (A and B) Uptake (A) and phagocytosis (B) of bacteria (*E. coli 25922GFP* and *L. monocytogenes*) in isolated neutrophils from bone marrow are expressed as MFI. Neutrophils were co-incubated with *E. coli 25922GFP* or CFSE-labeled live or heat-treated bacteria for 60 min in antibiotic-free medium. Next, cells were washed to remove non-phagocytosed bacteria. Phagocytosed bacteria were measured by flow cytometry. Representative FACS profiles are shown. The bar graphs underneath the FACS profiles show the mean ± SEM MFI value from one representative experiment (n = 3, cells from three male mice). The colors used in the bar graphs correspond to the colors of the lines in the FACS profiles. (C) Uptake and phagocytosis of bacteria (*DH5*α and *S. aureus*) in isolated neutrophils from bone marrow were expressed as MFI. (D and E) *In vitro* growth of *E. coli 25922* (D) and *L. monocytogenes* (E) in isolated neutrophils. Neutrophils were co-incubated with *E. coli 25922* or *L. monocytogenes* for 60 min in antibiotic-free medium and then gentamycin (100 μg mL^−1^) was added to the medium; neutrophils were collected after further 30-, 90-, 150-, 210-, and 270-min incubations, and the cells were lysed and plated to obtain the c.f.u. (n = 5). Data are represented as mean ± SEM from two (D and E) and three (A–C) independent experiments. Student's t test, ∗∗p < 0.01, ∗∗∗p < 0.001, n.s., not significant.

To elucidate the signaling mechanisms by which Siglec-E regulates bacterial infection, we examined bacterial clearance *in vitro* by using neutrophils isolated from wild-type littermates and Siglec-E-deficient mouse bone marrow (Figure S7). Neutrophils were co-incubated with *E. coli 25922GFP* or carboxyfluorescein succinimidyl ester (CFSE)-labeled live or heat-treated bacteria (Figure S8) for 60 min in antibiotic-free medium. Next, the medium was changed and the cells were washed with PBS to remove non-phagocytosed bacteria. Phagocytosed bacteria were measured by flow cytometry. The bacterial content was equal at this time as indicated by comparable MFI (Figures 2A–2C). Similar results were obtained for peritoneal macrophages (Figures S9 and S10), demonstrating equal uptake and phagocytic capacity in both genotypes.

We used a gentamicin-killing assay to investigate whether Siglec-E regulates bacterial clearance during infection. Neutrophils isolated from wild-type and Siglec-E-deficient mouse bone marrow were co-incubated with live bacteria for 60 min in antibiotic-free medium. The cells were collected after additional 30-, 90-, 150-, 210-, and 270-min incubations with medium containing gentamycin, and intracellular bacterial burdens were quantified. Wild-type neutrophils efficiently cleared bacteria, whereas bacterial content increased over time in Siglec-E-deficient neutrophils (Figure 2D). The two genotypes showed no differences in clearance of *L. monocytogenes* (Figure 2E).

These results suggest uptake and phagocytosis of Gram-positive *S. aureus* and *L. monocytogenes* and Gram-negative *E. coli 25922* and *DH5α* by Siglec-E-deficient neutrophils were comparable with those of wild-type neutrophils. However, Siglec-E participates in intracellular killing of ingested live Gram-negative bacteria but not Gram-positive *L. monocytogenes* as intracellular killing of *E. coli 25922* was markedly lower in Siglec-E-deficient neutrophils than in wild-type neutrophils. Therefore, a difference in bacterial clearance likely underlies the resistance of wild-type mice to Gram-negative bacterial infection.

### Tyr432 in the ITIM Domain of Siglec-E Is Critical for ROS Production

**Figure 3:**
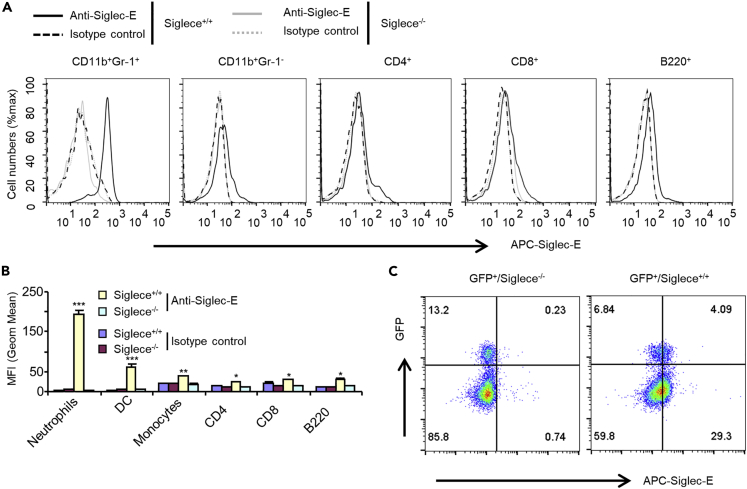
Expression of Siglec-E on Leukocyte Populations (A) Flow cytometric analysis of expression of Siglec-E on wild-type C57BL/6 mouse leukocytes from spleen. Representative FACS profiles are shown. Experiments in this figure were reproduced four times. (B) The bar graphs show the mean ± SEM MFI value from A (n = 3, cells from three male mice). Student's t test, ∗p < 0.05, ∗∗p < 0.01, ∗∗∗p < 0.001. (C) Flow cytometric analysis of expression of Siglec-E on regulatory T cells (Tregs). Spleens were collected from *Siglece*^*+/+*^*FOPX3*^*IRES-GFP*^ or *Siglece*^*−/−*^*FOPX3*^*IRES-GFP*^ mice and stained with anti-Siglec-E antibodies. Similar results were found when neutrophils were gated with CD111b^+^Gr-1^+^ or CD11b^+^Ly6G^+^ and monocytes were gated with CD111b^+^Gr-1^-^ or CD11b^+^Ly6C^+^ (data not shown). Representative FACS profiles are shown. Experiments in this figure were reproduced three times.

The surface expression of Siglec-E on immune cells was determined by flow cytometry. Consistent with published data (McMillan et al., 2013; Zhang et al., 2004), neutrophils showed the highest expression of Siglec-E, followed by monocytes, CD4^+^ T cells, CD8+ T cells, B cells, and a small population of regulatory T cells (Tregs) (Figure 3). These findings suggest Siglec-E may have an important role in these cells.

**Figure 4:**
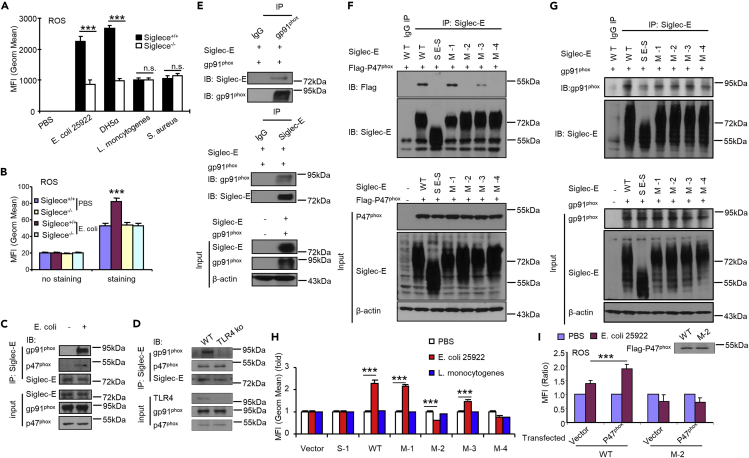
Tyr432 in the ITIM Domain of Siglec-E is Critical for ROS Production (A) ROS production after infection. Spleen cells were collected 16 h after infection and labeled with H_2_DCFDA at a final concentration of 10 μM at 37°C for 15 min. ROS production was measured using flow cytometric analysis. Neutrophils were gated with CD11b^+^ and GR-1^+^ (n = 5). (B) Neutrophils isolated from uninfected mice bone marrow were infected with *E. coli 25922* (MOI of 100:1) for 5 h at 37°C *in vitro*. ROS production was detected as in (A). (C) Siglec-E interacts with gp91^phox^ and p47^phox^ endogenously in neutrophils. Neutrophils isolated from *Siglece*^*−/−*^ or WT littermates bone marrow were treated with *E. coli 25922* (MOI of 100:1) or untreated for 5 h at 37°C *in vitro*. Siglec-E was immunoprecipitated (IP) with Siglec-E antibodies and blotted for gp91^phox^, p47^phox^, and Siglec-E antibodies. (D) Interaction between Siglec-E and gp91^phox^ is dependent on TLR4 activation. Neutrophils isolated from *Tlr4*^*−/−*^ or WT bone marrow were treated with *E. coli 25922* (MOI of 100:1) for 5 h at 37°C *in vitro*. Siglec-E was immunoprecipitated (IP) with Siglec-E antibodies and blotted for gp91^phox^, p47^phox^, TLR4, and Siglec-E antibodies. (E) Siglec-E interacts with gp91^phox^ and p47^phox^*in vitro*. IP and immunoblot analysis of the indicated proteins in HEK293T cells co-transfected with Siglec-E, gp91^phox^, and p47^phox^. (F and G) Mapping of the Siglec-E domain interacting with p47^phox^ (F) and gp91^phox^ (G) in HEK293T cells. (H) The ITIM domain in Siglec-E is critical for its effect on Gram-negative bacterial infection-induced ROS production. Raw264.7 cells were reconstituted to express mouse wild-type Siglec-E and mutants and then infected with *E. coli 25922* or *L. monocytogenes* for 60 min. Then, ROS production was measured (n = 3, three different clones were analyzed for each mutant). (I) The M-2 region in the ITIM domain is required for ROS production. Raw264.7 cells reconstituted to express mouse wild-type Siglec-E or M-2 were transfected with empty vector or FLAG-p47^phox^ for 48 h and infected with *E. coli 25922* for 60 min. Then, ROS production was measured. Inset shows equal amounts of FLAG-p47^phox^ expressed in these cells as determined by western blotting with anti-FLAG. Data are presented as mean ± SEM from two (A and B) and three (H) independent experiments. Experiments (C–G) were reproduced twice. Student's t test, ∗∗p < 0.01, ∗∗∗p < 0.001, n.s., not significant.

Neutrophils are the most abundant leukocytes in the blood and are crucial in immune responses against pathogens. Neutrophils produce several potent antimicrobial molecules like ROS and release cationic peptides, proteases, lactoferrin, and chromatin that form neutrophil extracellular traps to kill bacteria after encountering pathogens (Nguyen et al., 2017; Dahlgren et al., 2019). To further elucidate the molecular mechanisms of Siglec-E in bacterial clearance, we determined the production of ROS in neutrophils during infection. We used flow cytometry-based measurements after staining with 2′,7′-dichlorodihydrofluorescein diacetate (H_2_DCFDA, a cell-permeable indicator used to measure total intracellular ROS). We found *Siglece*^*−/−*^ neutrophils produced significantly lower levels of ROS than wild-type neutrophils during infection with Gram-negative *E. coli 25922* or *DH5α* but not with Gram-positive *S. aureus* or *L. monocytogenes* (Figure 4A). Neutrophils isolated from uninfected mice and then infected with *E. coli 25922 in vitro* showed similar results (Figure 4B).

Neutrophils contain a specialized enzyme system (NADPH oxidase) that enables ROS production. NADPH oxidase is a multicomponent enzyme consisting of membrane-bound gp91^phox^ and p22^phox^, together with cytoplasmic subunits (p47^phox^, p40^phox^, and p67^phox^). Thus, we investigated how Siglec-E regulates ROS production by controlling the activity of NADPH oxidase. Given their colocalization in the membrane, we determined whether Siglec-E interacts with the NOX2 complex. We found endogenous Siglec-E interacts with endogenous gp91^phox^ and p47^phox^ in neutrophils after treatment with *E. coli* (Figure 4C). The interaction between siglec-E and gp91^phox^ was dependent on the activation of TLR4, but the interaction between siglec-E and p47^phox^ was not (Figure 4D). This interaction was further explored using immunoprecipitation of cell lysates of HEK293T cells transfected with expression vectors for Siglec-E, gp91^phox^, and p47^phox^ (Figure 4E). Negative regulatory signaling by most Siglec proteins can be attributed to their immunoreceptor tyrosine-based inhibitory motif (ITIM) domains (Chen et al., 2014b). Thus, we made a short form of Siglec-E (SE-S: the cytoplasmic domain was deleted, including all ITIM domains in Siglec-E) and four Siglec-E point mutants, M-1 (R126D), M-2 (Y432F), M-3 (Y455F), and M-4 (both Y432F and Y455F), to map the site of association with p47^phox^ on Siglec-E. We co-transfected HEK293T cells with plasmids encoding wild-type or mutated Siglec-E and FLAG-p47^phox^. Immunoprecipitation was performed with antibodies for Siglec-E or FLAG. Mutation of Arg126 did not affect the binding ability of Siglec-E to p47^phox^; in contrast, mutation of Tyr455 partially affected the binding ability of Siglec-E to p47^phox^ (Figure 4F), but none of the mutants affected the interaction between Siglec-E and gp91^phox^ (Figure 4G). Both short forms of Siglec-E, M-2 and M-4, were unable to bind p47^phox^. Thus, we concluded Tyr432 on the ITIM domain in Siglec-E is critical for the interaction with p47^phox^.

We established Raw264.7 stable cell lines overexpressing different Siglec-E mutants to determine whether the ITIM domains of Siglec-E are required for ROS production. Overexpression of wild-type Siglec-E and mutant M-1 but not mutant M-2 and M-4 in RAW264.7 cells (Figure S11) significantly promoted ROS production (Figure 4H) during infection with *E. coli 25922* but not *L. monocytogenes*, indicating Tyr432 in the ITIM domain is required for ROS production. Consistent with mutation of the Tyr455 site partially affecting the binding ability of Siglec-E to p47^phox^ (Figure 4F), the production of ROS was also reduced with mutation of Y455F in Siglec-E (Figure 4H). As a control, overexpression of Siglec-1 had no effect on ROS production. Based on these findings, Siglec-E promotes production of ROS via Tyr432 in ITIM domain during bacterial infection. Overexpression of p47^phox^ Raw264.7 cells further confirmed theTyr432-dependent interaction between Siglec-E and p47^phox^ is required for ROS production (Figure 4I).

### Enhanced Monocyte and Neutrophil Recruitment in Gram-Negative but Not Gram-Positive Bacterial Infection in *Siglece*^*−/−*^ Mice

**Figure 5:**
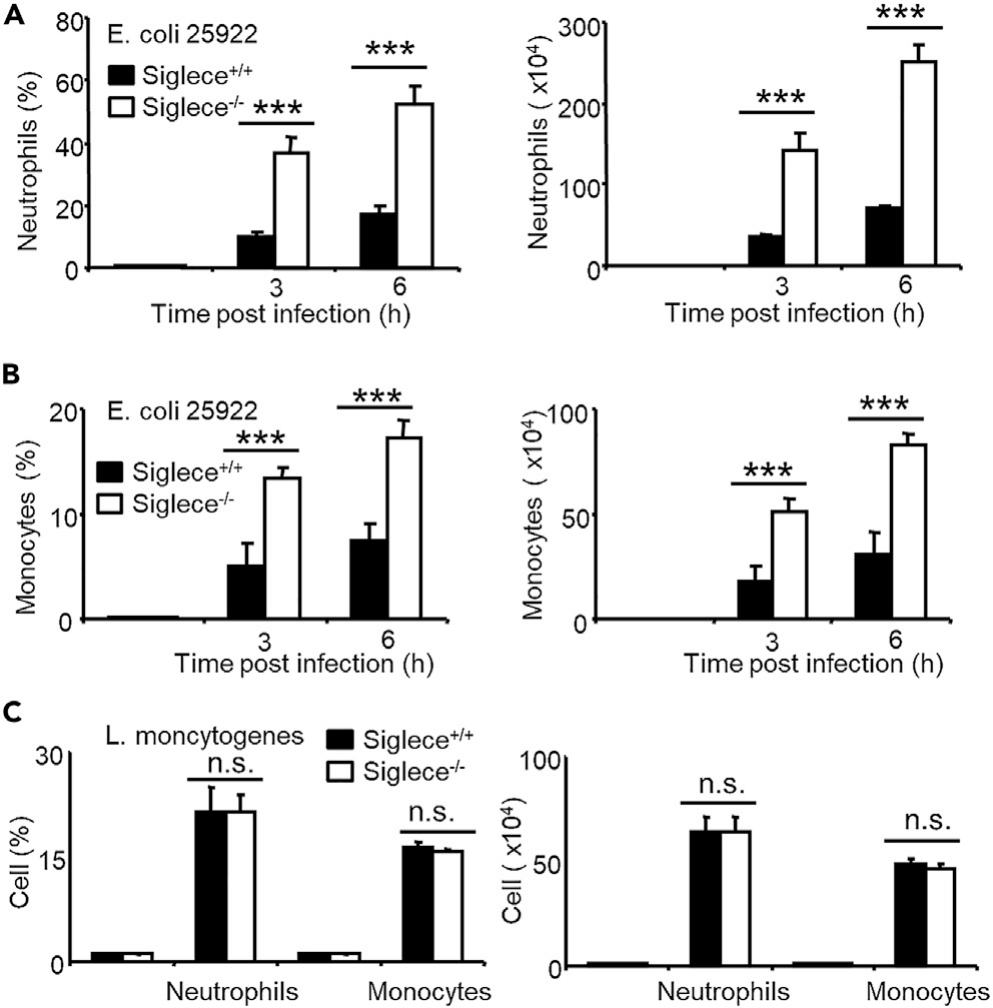
Enhanced Monocyte and Neutrophil Recruitment in Gram-Negative Bacterial Infected *Siglece*^*−/−*^ Mice (A and B) At 3 and 6 h after infection with *E. coli 25922*, peritoneal cells were collected and analyzed as CD11b^+^Gr-1^+^ (neutrophils) (A) and CD11b^+^Gr-1^-^ (monocytes) (B) cells. (C) Six hours after infection with *L. monocytogenes*, peritoneal cells were collected and analyzed as CD11b^+^Gr-1^+^ and CD11b^+^Gr-1^-^ cells. (n = 5). Student's t test, ∗∗∗p < 0.001, n.s., not significant.

Neutrophils develop in the bone marrow, and mature neutrophils egress into the circulation and migrate toward sites of infection to kill pathogens and remove cellular debris (Serbina and Pamer, 2006). Siglec-E reportedly controls neutrophil migration to the lungs following exposure to LPS. Thus, we determined whether Siglec-E also controls immune cell infiltration into the peritoneal cavity during bacterial infection. We measured immune cell infiltration in the peritoneal cavity of *E. coli 25922* and *L. monocytogenes*-infected animals by flow cytometry. Significantly higher infiltration of neutrophils and monocytes was observed in the peritoneal cavity of *E. coli 25922*-infected Siglec-E-deficient mice than in that of wild-type littermates (Figures 5A and 5B) but not in *L. monocytogenes*-infected mice (Figure 5C).

### Upregulation of Siglec-E Expression during Infection with Gram-Negative Bacteria

**Figure 6:**
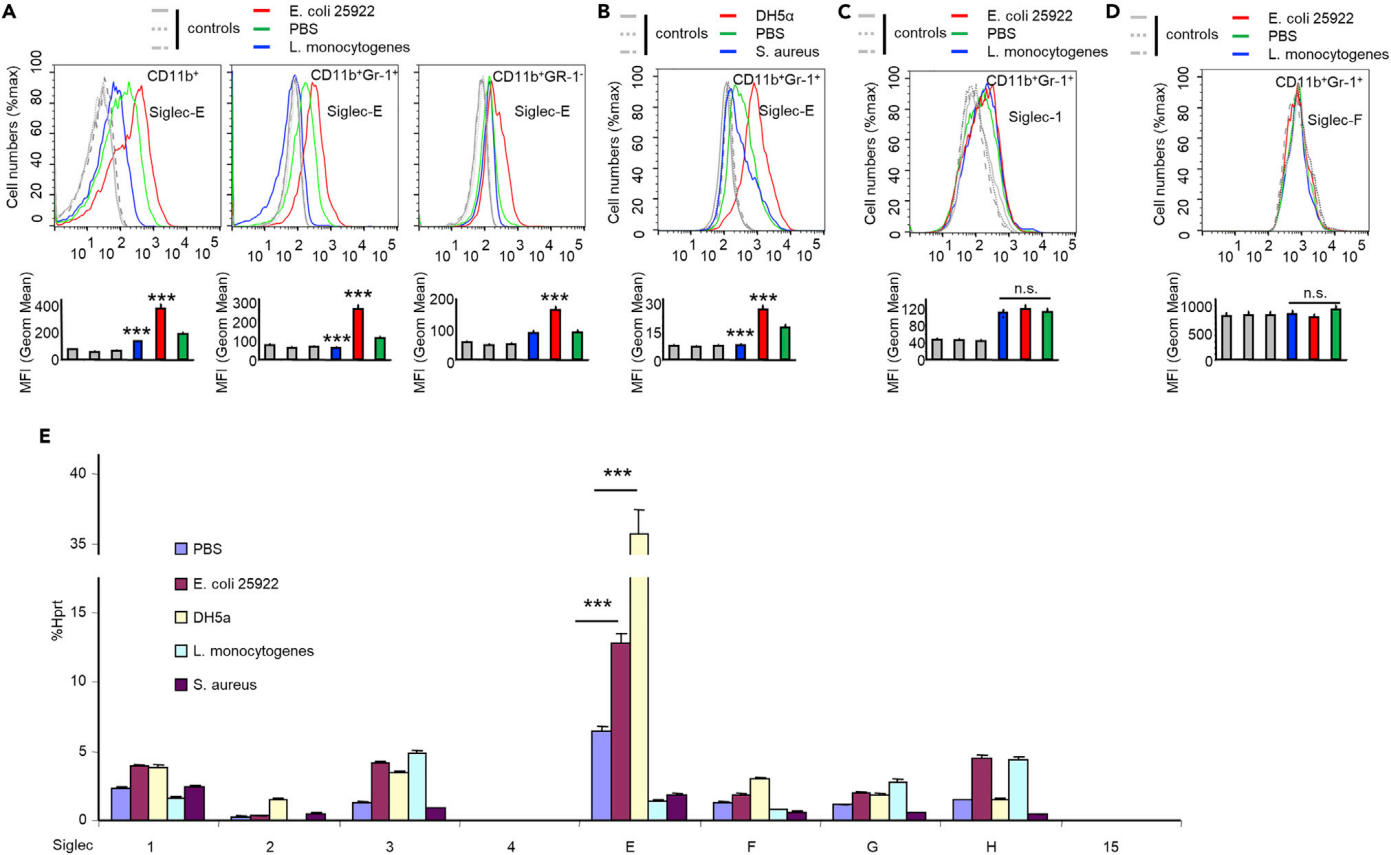
Differential Bacterial Clearance during Gram-Positive and Gram-Negative Bacterial Infection Is due to Differential Regulation of Siglec-E Expression (A–D) Flow cytometric analysis of Siglec expression. Spleen cells were collected at 16 h post infection (i.p. injection). Cell-surface Siglec-E (A and B), Siglec-1 (C), and Siglec-F (D) were determined by flow cytometric analysis. Representative FACS profiles are shown. The bar graphs underneath the FACS profiles show mean ± SEM MFI value from one representative experiment (n = 3, cells from three mice). The colors used in the bar graphs correspond to the colors of the lines in the FACS profiles. (E) Evaluation of Siglec expression in bacteria-infected-RAW264.7 cells by real-time PCR using Siglec-primer sets. Raw264.7 cells were infected with indicated bacteria (MOI = 100) for 5 h, and the expression of Siglecs was analyzed by real-time PCR. The bar graphs show mean ± SEM. Experiments in this figure were reproduced two (E) or three (A–D) times. Student's t test, ∗∗∗p < 0.001, n.s., not significant.

We sought to identify specific molecular mechanisms involved in the regulation of the innate response against Gram-negative bacterial infection. Therefore, we analyzed Siglec-E expression on splenic cells from mice infected with Gram-positive bacteria versus Gram-negative bacteria. Treatment with Gram-negative bacteria (*E. coli 25922*, *DH5α*) increased Siglec-E expression in splenic neutrophils and monocytes, whereas treatment with Gram-positive bacteria (*S. aureus*, *L. monocytogenes*) decreased expression of Siglec-E (Figures 6A and 6B). In contrast, Siglec-1 and Siglec-F were unaffected by infection with these bacteria (Figures 6C and 6D). Similar results were also obtained for mouse Raw264.7 cells (Figure 6E).

Moreover, Siglec-9, the human homolog of mouse Siglec-E, was upregulated in *E. coli 25922* infection and downregulated in *L. monocytogenes* infection in human monocytic cell line THP-1 (Figure S12A). Knockdown of Siglec-9 in THP-1 cells reduced ROS production during infection with *E. coli 25922* (Figures S12B and S12C). Taken together, these data suggest infection with Gram-negative bacteria *E. coli 25922* or *DH5α* upregulated Siglec-E expression in innate immune cells, whereas infection with Gram-positive bacteria *S. aureus* and *L. monocytogenes* downregulated Siglec-E but had no effect on Siglec-1 and F in innate immune cells.

### *E. coli 25922* Infection Upregulates Siglec-E Expression in Neutrophils via the TLR4/MyD88/JNK/NF-κB/AP-1 Signaling Pathway, whereas *L. monocytogenes* Infection Downregulates Siglec-E Expression in Neutrophils via the TLR2/RANKL/TRAF6/Syk Signaling Pathway

TLRs activate two distinct signaling pathways to control immune responses by recognizing conserved structures in pathogens: the MyD88-dependent and TRIF-dependent pathways (Wu et al., 2016b; Akira and Takeda, 2004; Liew et al., 2005; Kondo et al., 2012). The MyD88-dependent pathway is activated after the engagement of TLRs by their cognate PAMPs. For instance, after TLR4 encounters LPS, MyD88 recruits IL-1 receptor-associated kinases (IRAKs), which in turn activate NF-κB and MAPKs (Kondo et al., 2012; Kawai and Akira, 2010; Liew et al., 2005; Akira and Takeda, 2004). The TRIF-dependent pathway is triggered when TLR4 is delivered to endosomes and mediates activation of transcription factor IFN regulatory factor-3 (IRF3) through dimerization, which regulates type I IFN expression (Kondo et al., 2012; Kawai and Akira, 2010; Liew et al., 2005; Akira and Takeda, 2004).

**Figure 7:**
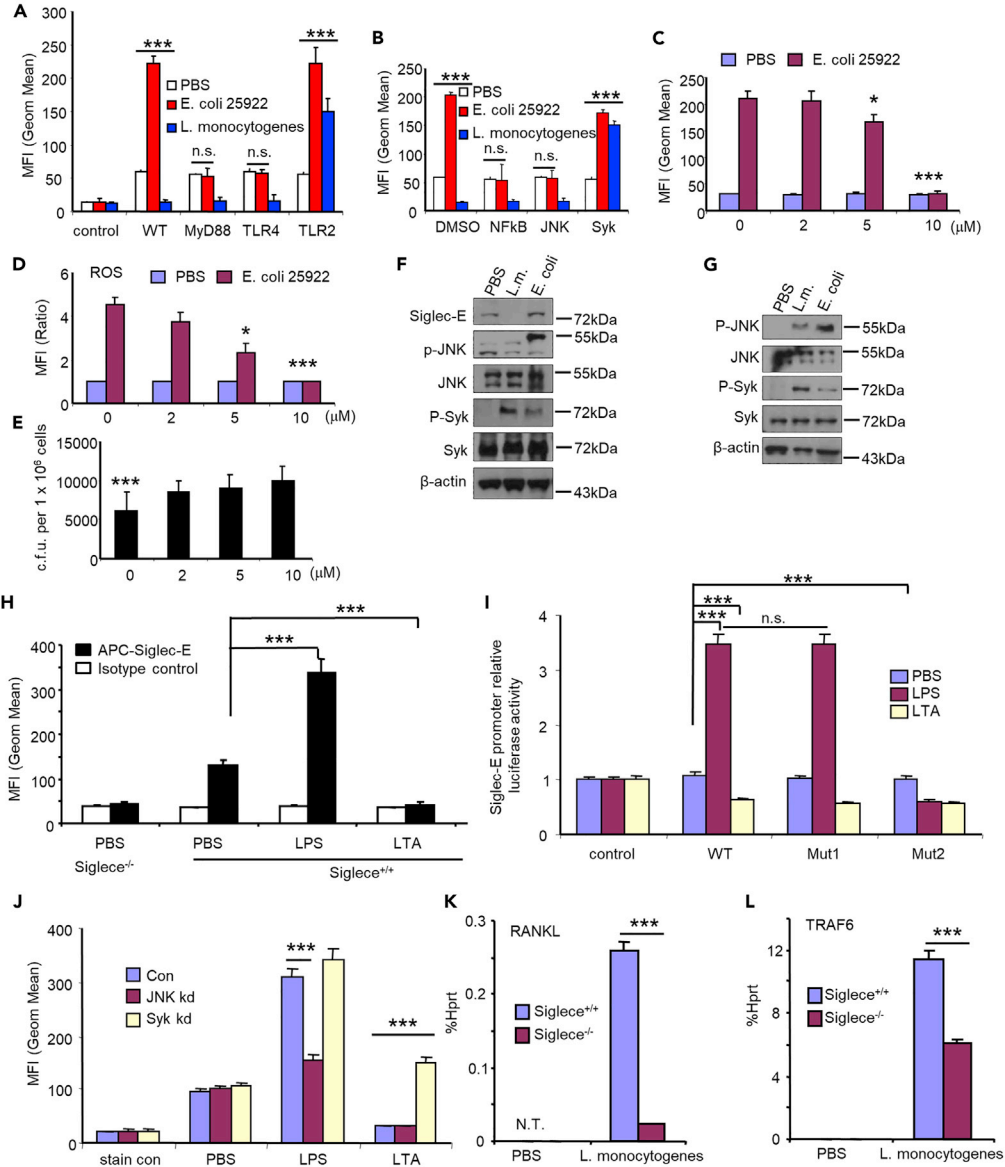
TLR4 Signaling Pathway Is Required for *E. coli 25922* Infection Induced-Siglec-E Upregulation, whereas TLR2 Signaling Pathway Is Required for *L. monocytogenes* Infection Induced-Siglec-E Downregulation. (A) Flow cytometric analysis of Siglec-E expression Wild-type and different knockout mice were i.p. injected with indicated bacteria. Spleen cells were collected 16 h after infection. Cell-surface Siglec-E was determined by flow cytometric analysis. The bar graphs show mean ± SEM MFI value from one representative experiment (n = 3, cells from three mice). Experiments in this figure were reproduced two times. (B) Flow cytometric analysis of Siglec-E expression after inhibitor treatment. Neutrophils were isolated from bone marrow. Cell-surface Siglec-E was determined by flow cytometric analysis 6 h post infection with or without inhibitors (Syk inhibitor piceatannol [75 μM], JNK inhibitor SP600125 [10 μM], NF-kB inhibitor Bay11-7085 [50 μM]). The bar graphs show the mean ± SEM MFI value from one representative experiment (n = 3, cells from three mice). Experiments in this figure were reproduced twice. Student's t test, ∗∗∗p < 0.001, n.s., not significant. (C–E) Raw264.7 cells were treated with different doses of JNK inhibitor SP600125 for 15 h and then infected with *E. coli 25922* or PBS for 1 h. The expression of Siglec-E (C) and ROS production (D) was determined by flow cytometry, and *in vitro* growth of *E. coli 25922* (E) was measured as in Figure 2D. (F and G) Immunoblot analysis of the indicated molecules in lysates of RAW264.7 cells (F) and isolated neutrophils from mouse bone marrow (G) 5 h after infection. E. coli, *E. coli 25922*; L.m., *L. monocytogenes*. Representative western blot images from two independent experiments. (H) Neutrophils were isolated from bone marrow and stimulated with LPS or LTA for 16 h. Cell-surface Siglec-E was determined by flow cytometric analysis. The bar graphs show the mean ± SEM MFI value from one representative experiment (n = 3, cells from three mice). Experiments in this figure were reproduced twice. Student's t test, ∗p < 0.05, ∗∗∗p < 0.001. (I) Luciferase activity in lysates of Raw264.7 cells co-transfected with PGL3 (control), PGL3-Siglec-E promoter (WT), or PGL-3-Siglec-E promoter mutants (Mut1, Mut2); luciferase reporter plasmids; and pTK-Renilla-luciferase plasmids for 24 h. Then, cells were stimulated with LPS or LTA for 16 h. Luciferase activity is presented relative to Renilla luciferase activity. Experiments in this figure were reproduced two times. Student's t test, ∗∗∗p < 0.001, n.s., not significant. (J) Cell-surface Siglec-E on RAW264.7 cells transfected with JNK or Syk siRNA for 48 h and then stimulated with LPS or LTA for 16 h. The bar graphs show the mean ± SEM MFI value from one representative experiment. Experiments in this figure were reproduced two times. Student's t test, ∗∗∗p < 0.001. Con, transfected with control siRNA; JNK kd, transfected with JNK siRNA; Syk kd, transfected with Syk siRNA. (K and L) Real-time PCR analysis of RANKL (K) and TRAF6 (L) expression in neutrophils. Neutrophils were purified from mouse bone marrow and then infected with *L. monocytogenes* (MOI = 100) for 5 h. Experiments in this figure were reproduced two times. Student's t test, ∗∗∗p < 0.001.

The mechanism underlying the regulation of Siglec-E expression during infection was further examined. Wild-type, TLR2, TLR4, and MyD88 knockout mice were infected with *E. coli 25922* or *L. monocytogenes* for 16 h, and the expression of Siglec-E or Siglec-1 or Siglec-F on spleen cells was determined by flow cytometry. The upregulation of Siglec-E observed in splenic neutrophils from wild-type mice infected with *E. coli 25922* was abolished in neutrophils from mice deficient in either MyD88 or TLR4 but not in those from TLR2 knockout mice (Figure 7A). Interestingly, infection with *L. monocytogenes* decreased Siglec-E expression in all groups except for TLR2-deficient neutrophils, which showed increased expression (Figure 7A). To understand the signaling mechanisms through which bacterial infections regulate Siglec-E expression, we first tested whether a protein kinase may modulate *E. coli*-induced Siglec-E expression. We isolated neutrophils from wild-type mouse bone marrow and pretreated with NF-κB or JNK or Syk inhibitors prior to infection with *E. coli 25922* or *L. monocytogenes*. Treatment with NF-κB and JNK inhibitors abolished *E. coli 25922*-induced upregulation of Siglec-E expression, whereas Syk inhibitor rescued *L. monocytogenes*-induced downregulation of Siglec-E expression (Figure 7B). JNK inhibitor abolished *E. coli 25922*-induced upregulation of Siglec-E expression in a dose-dependent manner (Figure 7C). Correspondingly, ROS production was deceased (Figure 7D) and bacterial growth was increased (Figure 7E). Accordingly, *E. coli 25922* infection triggered phosphorylation of JNK, whereas *L. monocytogenes* infection triggered phosphorylation of Syk in Raw264.7 cells and neutrophils isolated from mouse bone marrow (Figures 7F and 7G).

To determine why infection with Gram-negative bacteria induced the expression of Siglec-E but infection with Gram-positive bacteria reduced the expression of Siglec-E, we tested the effects of TLR4 ligand LPS and TLR2 ligand LTA on Siglec-E expression in neutrophils isolated from wild-type mouse bone marrow (Figure 7H). LPS treatment induced Siglec-E expression, whereas LTA treatment reduced Siglec-E expression (Figure 7H). Next, we determined whether Siglec-E was upregulated by signaling events downstream of TLR4/MyD88/JNK/NF-κB/AP-1. We found two AP-1 sites located 710 and 740 bp upstream of the translational start site of Siglec-E (Figure S13). We constructed luciferase reporters driven by the Siglec-E promoter containing a wild-type or mutated AP-1 site. Wild-type Siglec-E promoter-driven luciferase activity significantly increased in Raw264.7 cells treated with LPS, but promoter activity significantly decreased after LTA treatment (Figure 7I). Moreover, disruption of the AP-1 site in Mut2 but not Mut1 led to a complete elimination of LPS-induced promoter activities but had no effect on LTA treatment (Figure 7I). This finding suggests the AP-1 site in Mut2 is critical for the upregulation of Siglec-E expression during infection with Gram-negative bacteria.

The mechanisms underlying Siglec-E downregulation during infection with *L. monocytogenes* was further investigated. To confirm the results from the inhibitor studies (Figure 7B), we created JNK and Syk knockdown Raw264.7 cell lines using siRNA (Figure S14). We treated JNK and Syk knockdown Raw264.7 cells with LPS or LTA. As shown in Figure 7J, JNK knockdown blocked LPS-induced upregulation of Siglec-E expression and Syk knockdown restored LTA-induced downregulation of Siglec-E expression in Raw264.7 cells. RANKL and TRAF6 are regulators of *L. monocytogenes* infection via the TLR2 pathway, and upregulated RANKL and TRAF6 reduce phosphorylation of Syk (Leite, 2014; Konno et al., 2009; Knoop et al., 2009). Real-time PCR revealed the expression of RANKL and TRAF6 was significantly increased after infection with *L. monocytogenes* on neutrophils isolated from mouse bone marrow (Figures 7K and 7L).

These results suggest *E. coli* infection upregulates Siglec-E expression in neutrophils via the TLR4/MyD88/JNK/NF-κB/AP-1 signaling pathway, whereas infection with *L. monocytogenes* downregulates Siglec-E expression in neutrophils via the TLR2/RANKL/TRAF6/Syk signaling pathway.

## Discussion

Siglecs are sialic acid-binding immunoglobulin-like lectins and are differentially expressed on various subsets of leukocytes where they participate in the positive and negative regulation of immune and inflammatory responses in different medical conditions (Crocker and Redelinghuys, 2008). Most Siglecs inhibit immune responses via the recruitment of tyrosine phosphatases such as SHP1 and SHP2 by their cytoplasmic ITIM domain (Pillai et al., 2012). The role of Siglecs in infection with sialylated pathogens has been studied extensively. Nonetheless, how Siglecs respond to unsialylated bacterial infection remains unclear. We need to know how Siglecs respond to unsialylated bacterial infection since most pathogens are unsialylated (Chang and Nizet, 2014; Chen et al., 2014b). Here, Siglec-E expression was selectively upregulated during infection with Gram-negative bacteria *E. coli 25922* and *DH5α* but downregulated during infection with *S. aureus* and *L. monocytogenes*. In contrast, Siglec-1 and F were unaffected. Siglec-E is critical in many immune processes, including binding to the sialylated Tehuantepec strain (Erdmann et al., 2009), negatively regulating neutrophil recruitment into the lung (McMillan et al., 2013, 2014), and controlling the antiviral response (Boyd et al., 2009) and endocytosis (Wu et al., 2016b). By showing a critical role in controlling ROS production, we extended the function of Siglec-E in bacterial clearance during infection with unsialylated bacteria.

Sepsis is systemic inflammation occurring in response to infection. Despite the availability of antibiotics, hospitalization of patients with severe sepsis (septic shock) has increased rapidly (40% increase from 2012 to 2018) (Buchman et al., 2020; Walkey et al., 2015). Sepsis mortality rates remain high at ~30% (Lyle et al., 2014; Jawad et al., 2012; Buchman et al., 2020), causing approximately 200,000 deaths annually in the United States alone (Dombrovskiy et al., 2007; Buchman et al., 2020). Most cases of septic shock are caused by Gram-negative bacteria, and *E. coli* remains one of the most common pathogens leading to sepsis (McClean et al., 1994; Bosscha et al., 1997; Parrillo et al., 1990). This research indicates the immune regulatory processes that respond to sepsis are incompletely understood. Thus, further research is needed to decipher the immunological activity induced by sepsis. In this study, compared with wild-type littermates, *Siglece*^*−/−*^ mice were more susceptible to death following infection with Gram-negative bacteria (*E. coli 25922* and *DH5α*) but not with Gram-positive bacteria (*S. aureus* and *L. monocytogenes*). This result is due to the differential regulation of Siglec-E expression during infection with different bacteria. Infection with *E. coli 25922* and *DH5α* induced Siglec-E expression, whereas infection with *S. aureus* and *L. monocytogenes* reduced Siglec-E expression. In addition, rapid bacterial clearance is a fundamental determinant of outcomes in sepsis. We discovered Siglec-E controls neutrophil recruitment and regulates bacterial clearance by controlling ROS production through an interaction with gp91^phox^ and p47^phox^. Hence, Siglec-E might be a target in the treatment of patients with sepsis.

Siglec-E reportedly contributes to positive and negative regulation of ROS generation in different medical conditions, including inflammatory diseases, neurodegeneration, and cancer (Claude et al., 2013; Laubli et al., 2014; McMillan et al., 2014; Schwarz et al., 2015). Siglec-E on microglia inhibited phagocytosis of neuronal debris and prevented the production of superoxide radicals induced by neuronal debris (Claude et al., 2013). In contrast, Siglec-E promoted ROS production in neutrophils in response to fibrinogen *in vitro* (McMillan et al., 2014). Pre-activation of neutrophils with phorbol 12-myristate 13-acetate (PMA) increased production of ROS in Siglec-E-deficient neutrophils (Laubli et al., 2014). Siglec-E receptors can also impact mammalian lifespan by modulating oxidative stress (Schwarz et al., 2015). In addition, Siglec-9 (human homolog Siglec-E) negatively regulated ROS production during infection with Group B *Streptococcus* (GBS) (Carlin et al., 2009). However, the mechanism underlying Siglec-E-mediated ROS production is unclear. Here we found Siglec-E controls ROS production through an interaction with gp91^phox^ and p47^phox^. Additionally, Tyr432 on the ITIM domain in Siglec-E is critical for the interaction with p47^phox^.

Siglec-E is mainly expressed on neutrophils. Neutrophils participate in the response to bacterial infection by producing several potent antimicrobial molecules like ROS and releasing cationic peptides, proteases, lactoferrin, and chromatin that form neutrophil extracellular traps (NETs) to kill bacteria after encountering pathogens. We show Siglec-E controls bacterial infections through regulating bacterial clearance by binding to gp91^phox^ and p47^phox^ to maintain the stability of the NOX2 complex, thereby promoting ROS production. During infection, neutrophils produce ROS to kill bacteria. However, their potential to form NETs, an anti-microbial defense mechanism that clears microorganisms, is compromised. Therefore, further investigation is required to determine whether Siglec-E plays a role in NET formation during infection with unsialylated bacteria.

In sepsis induced by Gram-negative bacteria, LPS from Gram-negative bacteria, CD14, and TLR4 form a complex to activate several intracellular signaling pathways including NF-κB, MAPKs (such as p38), JNK, and Erk. In turn, these components synergize while activating transcriptional factors AP-1 and IRF3, which control the expression of immune genes and production of cytokines (Akira and Takeda, 2004; Liew et al., 2005; Kondo et al., 2012; Kawai and Akira, 2010). We show Gram-negative bacterial infection upregulates Siglec-E expression via the TLR4/MyD88/JNK/NF-κB/AP-1 signaling pathway, whereas infection with Gram-positive bacteria downregulates Siglec-E expression via the TLR2/RANKL/TRAF6/Syk signaling pathway.

Our study describes a new role for Siglec-E during infection. We demonstrate genes intricately regulated during host-pathogen interactions. Enhanced Siglec-E expression dampens the innate response to Gram-negative bacterial infection. In contrast, Gram-positive bacteria avoid host defenses by repressing Siglec-E expression. Moreover, inhibition of Siglec-E expression by inhibitors or Siglec-E antibodies will reduce ROS production but induce neutrophil migration. Thus, Siglec-E is a potential target for future treatments of patients with sepsis.

### Limitations of the Study

Here, we determined Siglecs respond to nonsialylated Gram-negative bacteria (*Escherichia coli 25922* and *DH5α*) and Gram-positive bacteria (*Staphylococcus aureus* and *Listeria monocytogenes*). Siglece^−/-^ mice had higher mortality than wild-type mice following Gram-negative but not Gram-positive bacterial infection. Although most microbial pathogens are nonsialylated, some oropharyngeal pathogens express sialic acid units on their surface. Therefore, further analyses using bacteria with sialic acid residues as controls are necessary to determine if Siglece^−/-^ mice show any change in mortality compared with wild-type mice following sialylated bacterial infection. Remaining questions include: are the bacteria carrying sialylated glycans efficiently cleared by Siglecs? Do these bacteria affect the expression of Siglecs? In addition, several glycans carry sugar residues that are very similar to sialic acid, such as deaminated neuraminic acid (KDN). How do Siglecs respond to infection with bacteria carrying these glycans?

### Resource Availability

#### Lead Contact

Further information and requests for reagents should be directed to the Lead Contact, Guo-Yun Chen (Gchen14@uthsc.edu).

#### Materials Availability

Materials are available from the Lead Contact upon reasonable request, but a Material Transfer Agreement may be required.

#### Data and Code Availability

The data that support the findings of this study are available from the Lead Contact upon request.

## Methods

All methods can be found in the accompanying Transparent Methods supplemental file.

## Supplemental Information

Document S1. Transparent Methods and Figures S1–S14
