# Supplementary material for: Selective Response to Bacterial Infection by Regulating Siglec-E Expression
Source: iScience. 2020 Aug 20;23(9):101473. doi: 10.1016/j.isci.2020.101473 (PMC7479279; doi:10.1016/j.isci.2020.101473)
Supplement: Document S1. Transparent Methods and Figures S1–S14 [file mmc1.pdf]

iScience, Volume *23*

Supplemental Information

Selective Response to Bacterial Infection by Regulating Siglec-E Expression

Yin Wu, Darong Yang, Runhua Liu, Lizhong Wang, and Guo-Yun Chen

Figure S1. Characterization of Siglec-E knockout mice, Related to Figure 1.

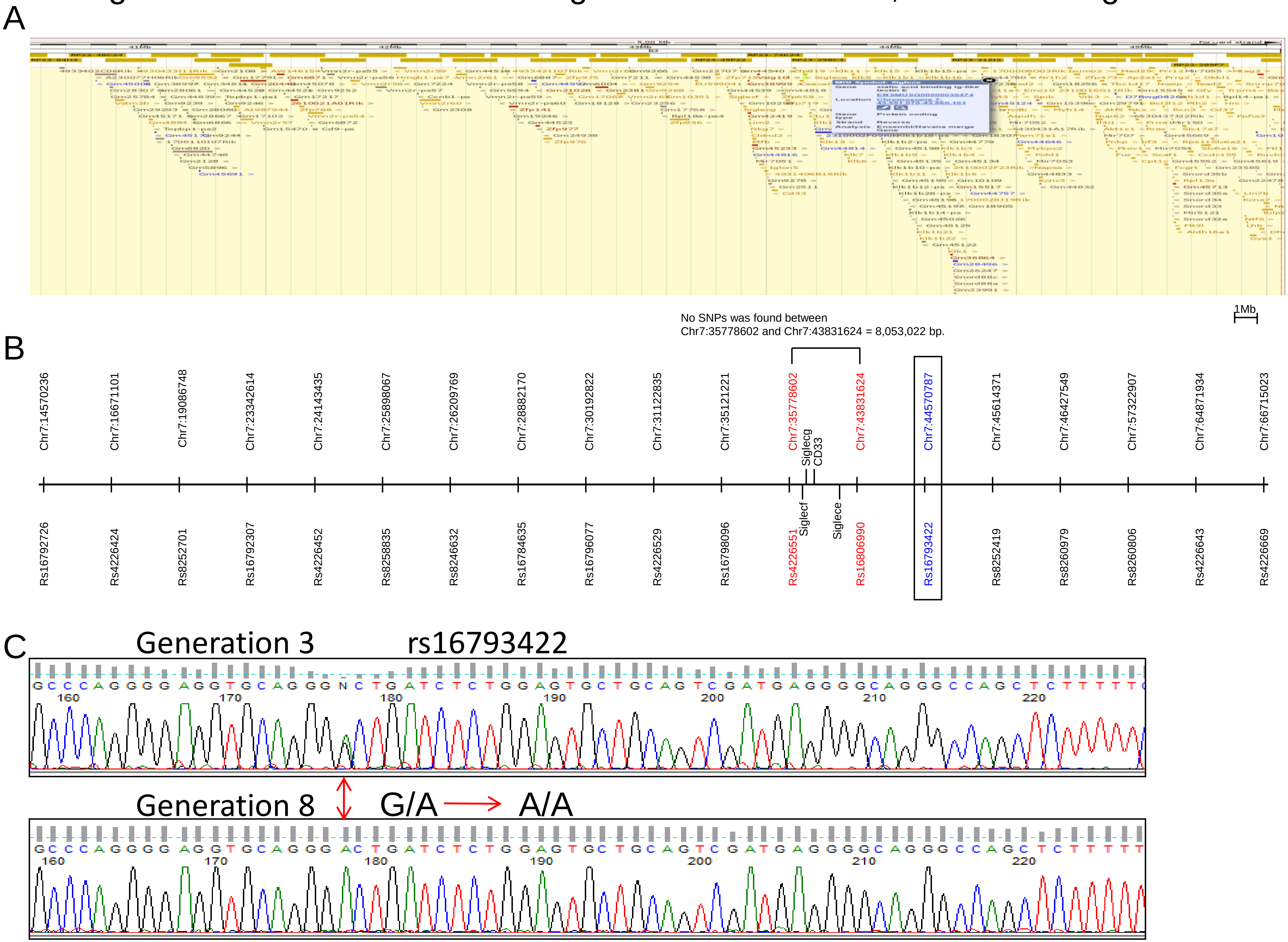

Figure S2. Determination of the sialylation level, Related to Figure 1 and 2.

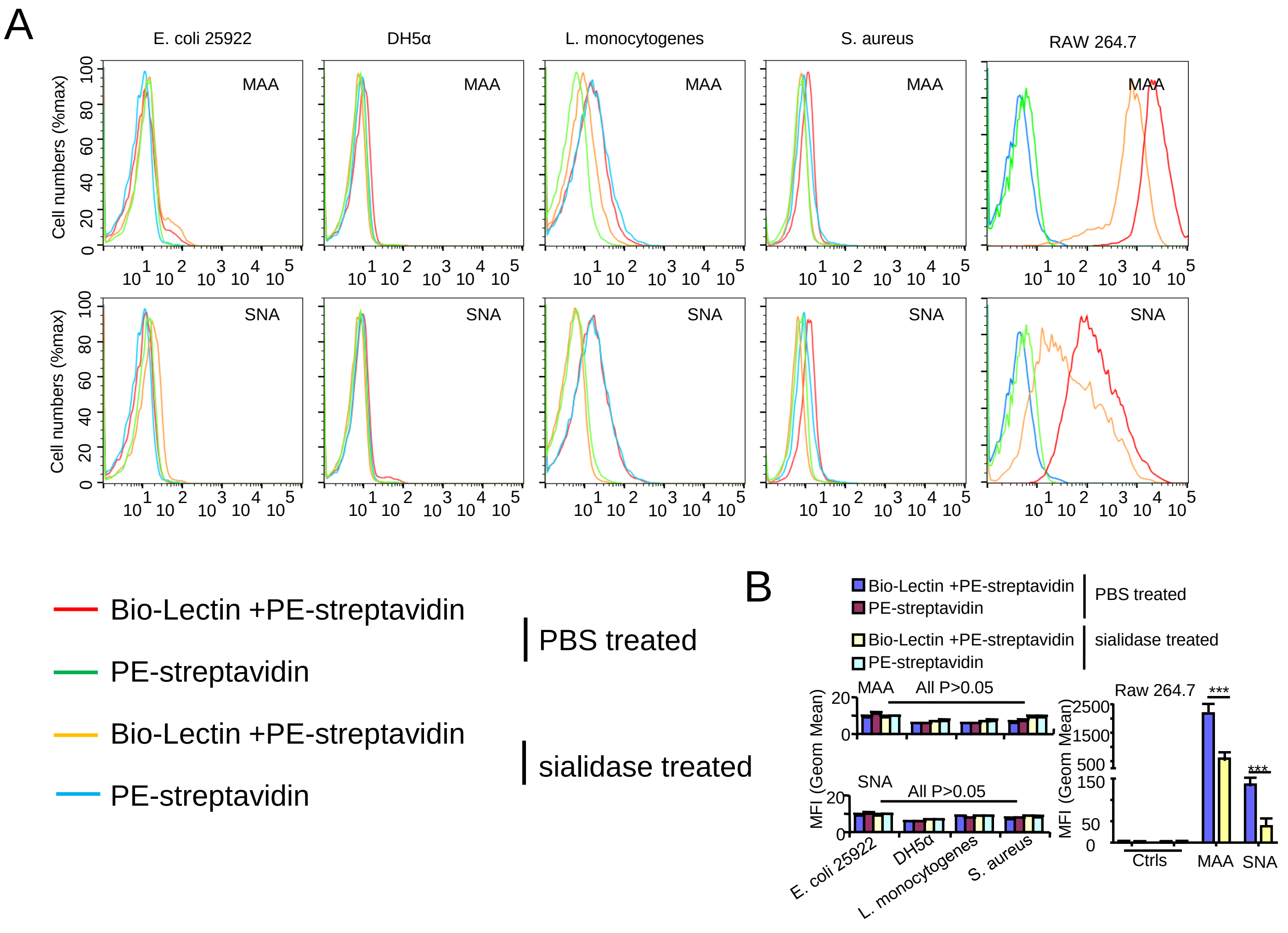

Figure S3. Flow cytometric analysis of bacterial interaction with Siglec-E, Related to Figure 1 and 2.

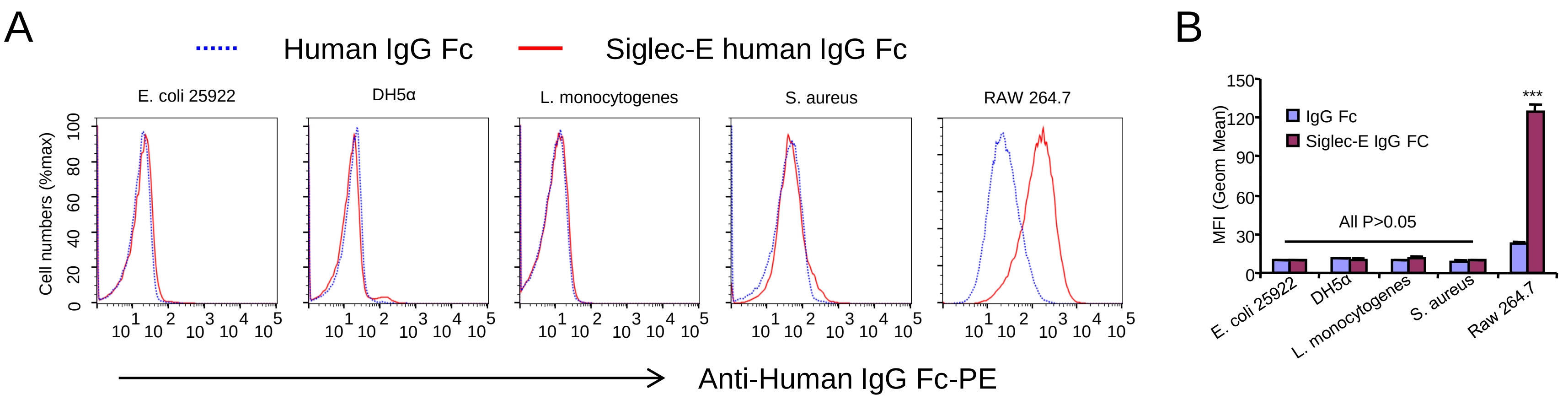

Figure S4. Mice survival analysis after LPS challenge, Related to Figure 1.

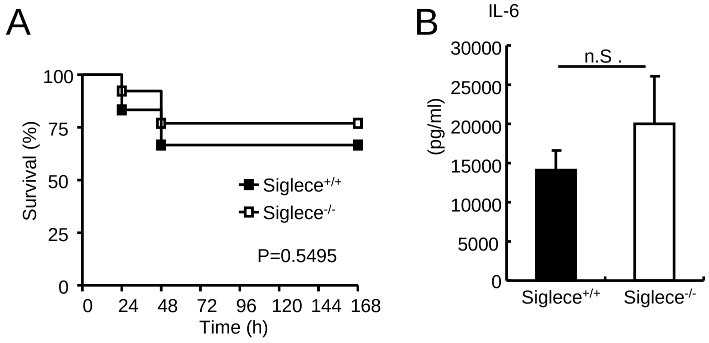

Figure S5. Cytokine production after bacterial infection, Related to Figure 1.

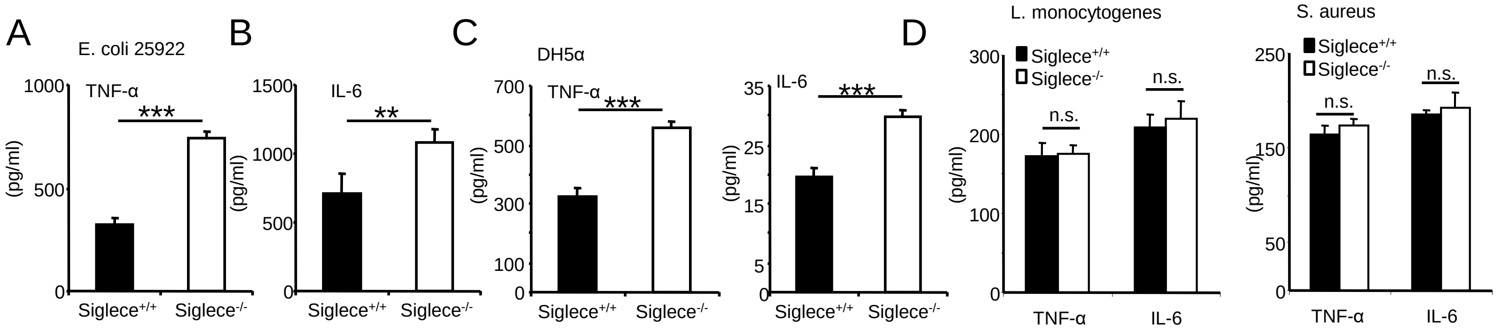

Figure S6. Siglec-F has no effect on bacterial clearance, Related to Figure 1.

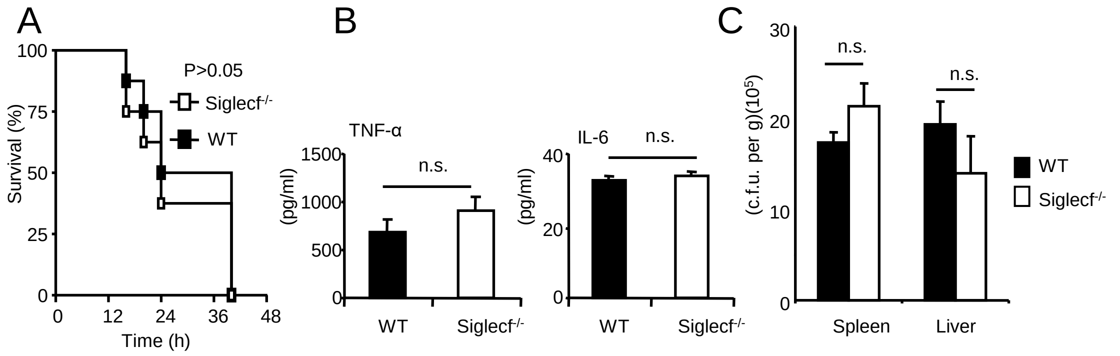

Figure S7. Flow cytometric analysis of neutrophils isolated from mouse bone marrow, Related to Figure 2.

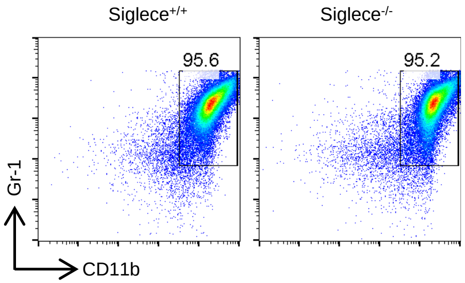

Figure S8. Flow cytometric analysis of bacteria labeled with CFSE (A), *E. coli 25922GFP* (B) , Related to Figure 2.

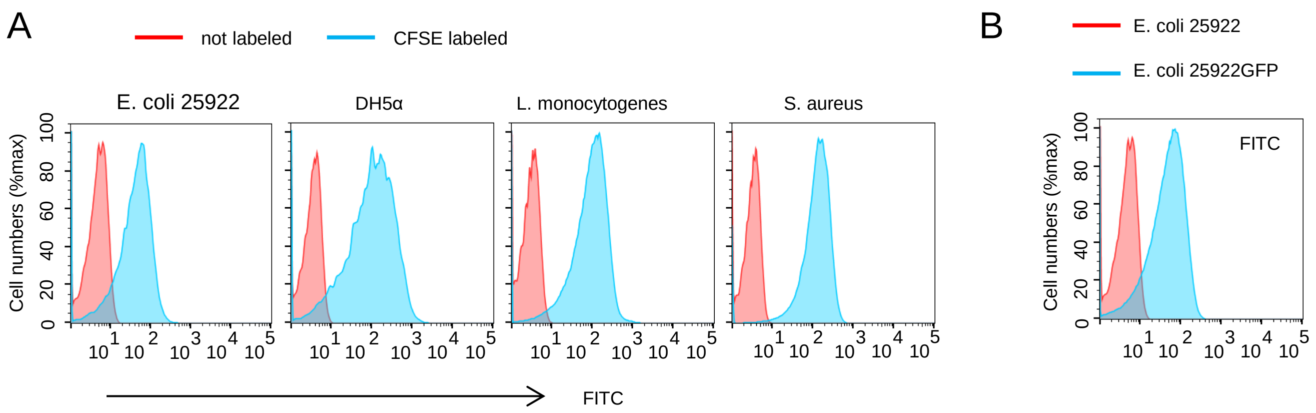

Figure S9. Flow cytometric analysis of uptake and phagocytosis of bacteria in peritoneal macrophages, Related to Figure 2.

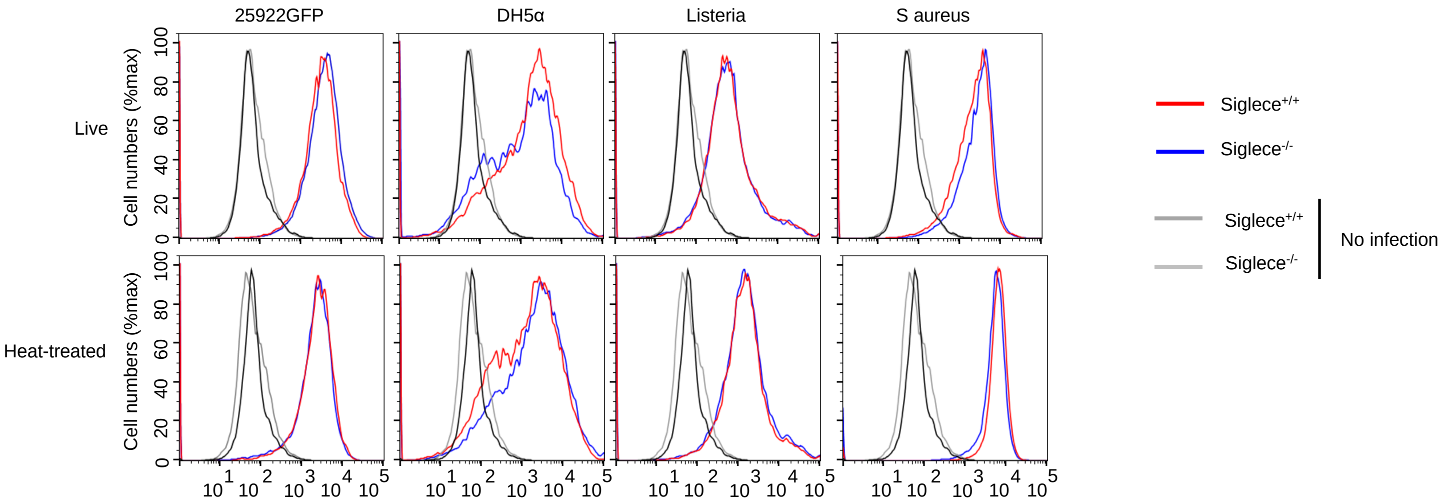

Figure S10. Flow cytometric analysis of uptake and phagocytosis of bacteria in Trypan treated or untreated peritoneal macrophages after bacterial infection, Related to Figure 2.

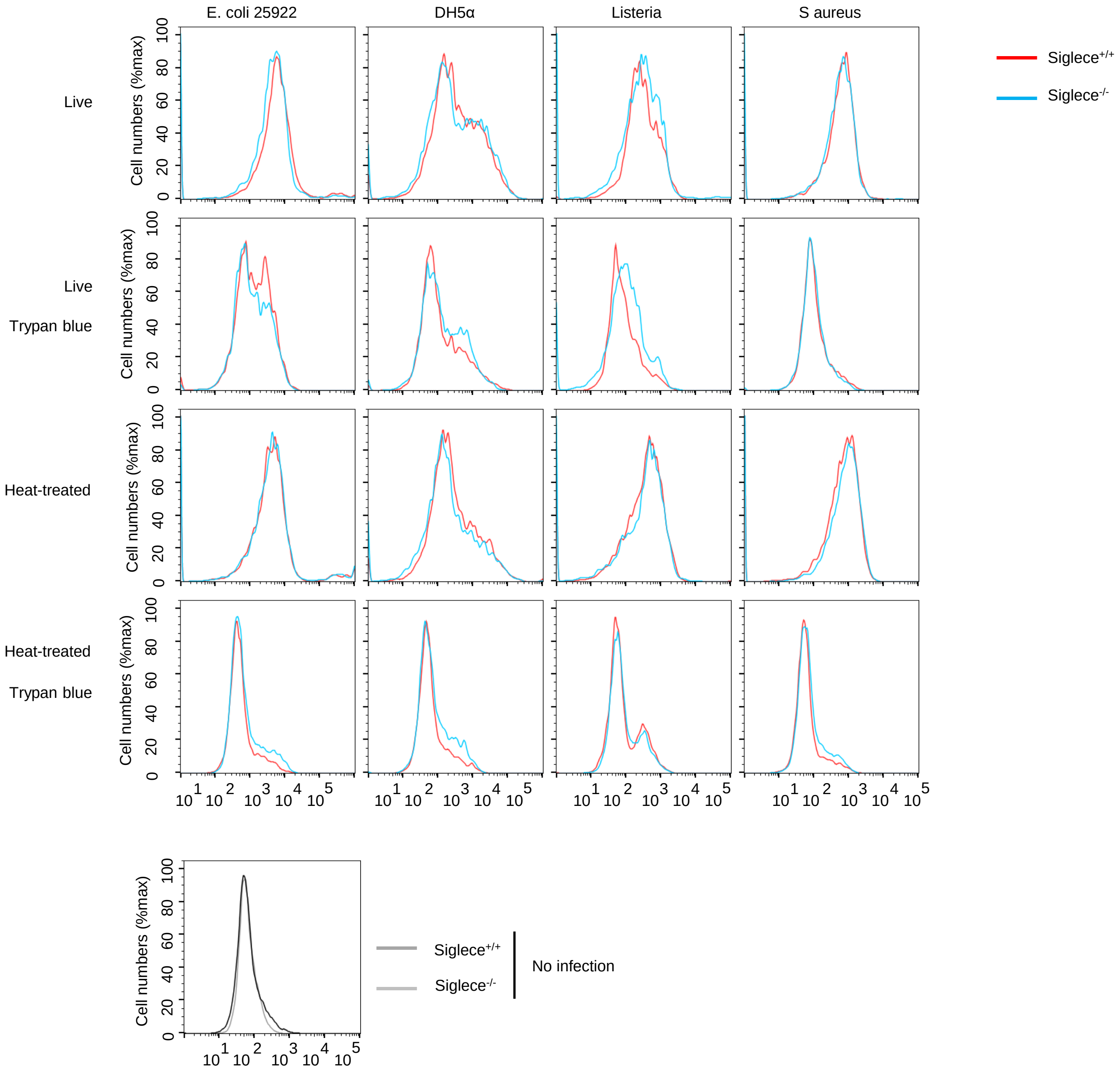

Figure S11. Establishing stable cell lines expressing of Siglec-E mutants in Raw264.7 cells, Related to Figure 4.

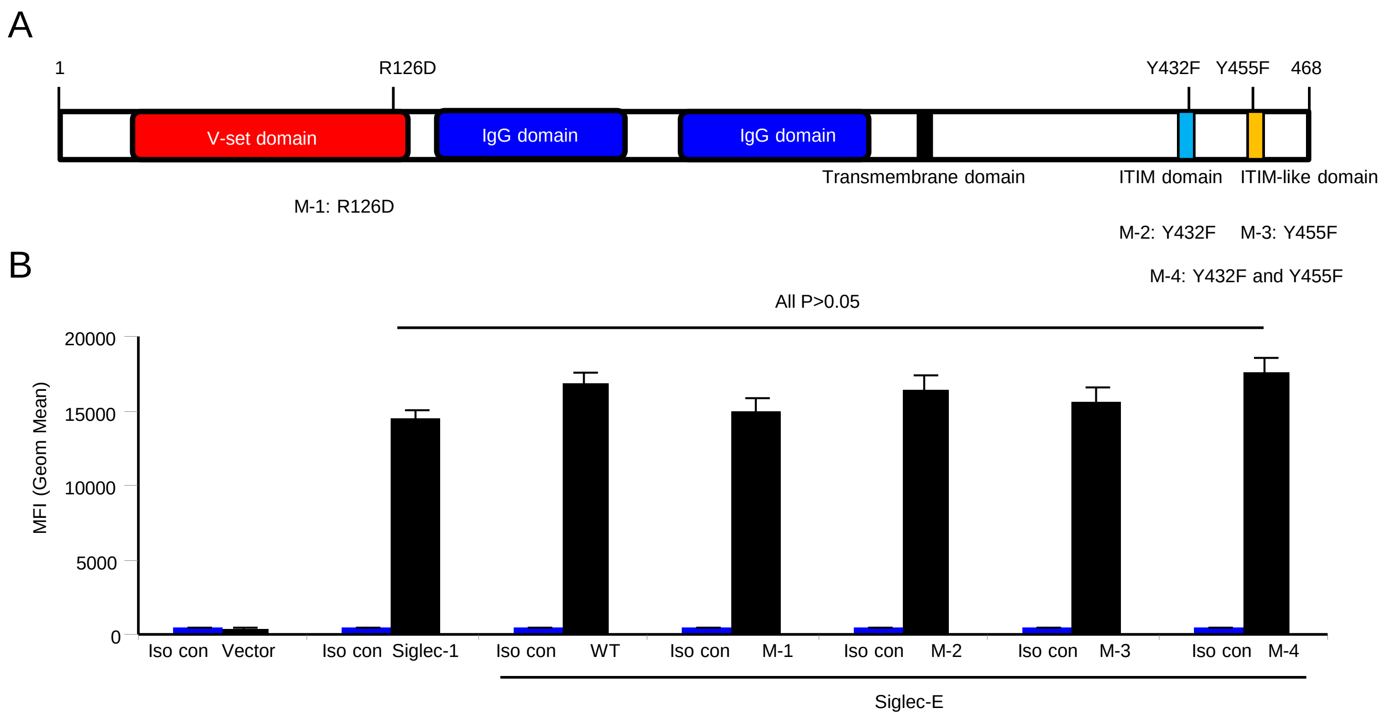

Figure S12. Evaluation of Siglec expression in bacteria infected-THP-1 cells by real-time PCR using Siglec primer sets, Related to Figure 6.

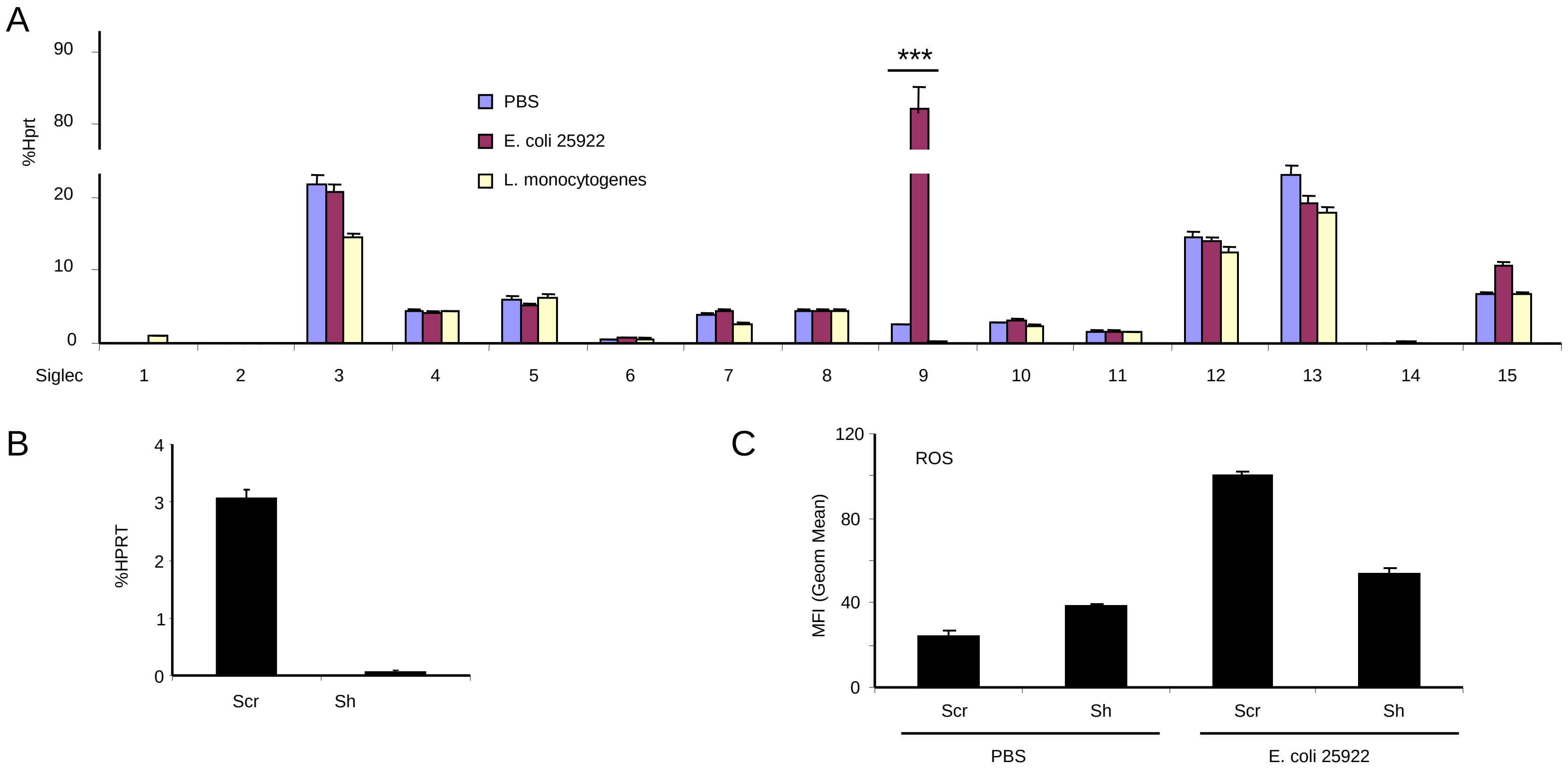

Figure S13. Siglec-E promoter and its putative AP-1 binding sites at 710 bp and 740 bp upstream of the translational start site (as +1) , Related to Figure 7.

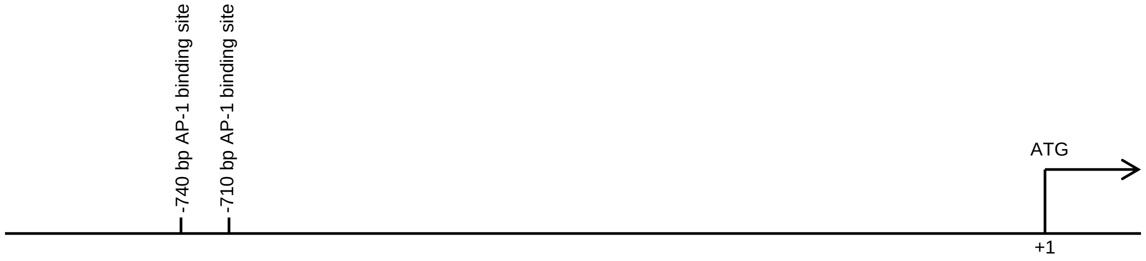

-740 -710

WT: CCCCCACTTCCTTTCTCTGTCTCCCTGTCCCTAGCTGGGAGGAAGTCCAGTCCTATTTTCTCCCCGACTCAGTCATTGACTGATCAGCTTCTTTATTGACCAATCAGGGAATAATTGGGT

Mut1: CCCCCACTTCCTTTCTCTGTCTCCCTGTCCCTAGCTGGGAGGAAGTCCAGTCCTATTTTCTCCCCGACaCAaaCATTGACTGATCAGCTTCTTTATTGACCAATCAGGGAATAATTGGGT

Mut2: CCCCCACTTCCTTTCTCTGTCTCCCTGTCCCTAGCTGGGAGGAAGTCCAGTCCTATTTTCTCCCCGACTCAGTCATTGACTGATCAGCTTCTTTATTGgCCAATCAGGGAATAATTGGGT

Figure S14. Evaluation of JNK and Syk expression in Raw264.7 cells, Related to Figure 7.

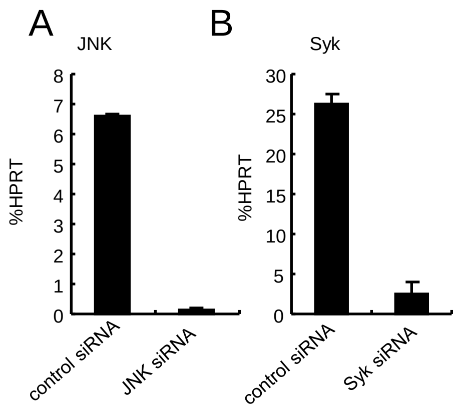

FIGURE LEGENDS

Figure S1. Characterization of Siglec-E knockout mice, Related to Figure 1. A, Siglec-E localization in Chromosome 7. **B**, Single-nucleotide polymorphism (SNPs) found between C57BL/6 and 129/Sv in the genomic DNA sequence region from Chr7:14570236 to Chr7:66715023 (within 100 mb of *Siglece*) were randomly selected as indicated for confirmation by PCR-sequencing. **C**, SNP rs16793422 G/A at generation 3 was backcrossed into A/A at generation 8.

Figure S2. Determination of the sialylation level, Related to Figure 1 and 2. *E. coli 25922* and *DH5α* were grown overnight in Luria-Bertani broth, collected by centrifugation at 1000 x g for 15 min and then washed twice with cold 1 x PBS. *Listeria monocytogenes* and *Staphylococcus aureus* were obtained from ATCC and propagated according to the manufacturer's protocol. **A, B**, Bacteria were treated with PBS or 1 unit/ml sialidase (37°C for one hour in PBS) and then stained with biotin-conjugated *Maackia amurensis* lectin I (MAA) (1 µg/ml, B-1265) recognizing α2-3–linked terminal sialic acid or biotin-conjugated *Sambucus nigra* (elderberry) bark lectin (SNA) (1 µg/ml, B-1305) recognizing α2-6–linked terminal sialic acid. Then, bacteria were detected with PE-Streptavidin. **A**, Representative FACS profiles are shown. **B**, The bar graphs show the mean ± S.E.M MFI value from one representative experiment (n = 3, cells from three mice). Statistical analysis was performed using two-tailed Student's t test. ***p < 0.001.

Figure S3. Flow cytometric analysis of bacterial interaction with Siglec-E, Related to Figure 1 and 2. **A**, **B**, Bacteria were stained with Siglec-E Fc (1 µg/ml, R & D, 5806-SL-050) or mouse IgG Fc (1 µg/ml) as a negative control and then detected with PE-anti-mouse IgG Fc. To prevent the internalization of MAA, SNA, Siglec E Fc, FITC-Streptavidin or PE-anti-mouse IgG

Fc by bacteria, all the staining steps were performed on ice for one hour. **A**, Representative FACS profiles are shown. **B**, The bar graphs present means ± SEM of mean fluorescence intensities (MFIs) pooled from three independent experiments. Statistical analysis was performed using two-tailed Student's t test. n.s., not significant. ***p < 0.001. Raw264.7 as a positive control. Experiments in this figure were reproduced three times.

Figure S4. Mice survival analysis after LPS challenge, Related to Figure 1. (**A**) Kaplan-Meier curve for female mice treated with LPS (200 µg, i.p. injection). Log-rank (Mantel-Cox) test. (n=14 for *Siglece*$^{+/+}$ mice, n=13 for *Siglece*$^{-/-}$ mice, combined from two independent experiments) (**B**) IL-6 in serum 16 h after LPS treatment. (n=5), Data shown are the means ± S.E.M. Student's t-test, n.s., not significant. Experiments in this figure were reproduced two times.

Figure S5. Cytokine production after bacterial infection, Related to Figure 1. Serum concentration of TNF-α and IL-6 in mice after i.p. injection for 16 h with *E. coli 25922* (n=5) (**A**), *DH5α* (n=5) (**B**), *S. aureus* (n=5) (**C**) and *L. monocytogenes* (n=5) (**D**). Data are represented as mean ± S.E.M from two independent experiments, Student's t-test, **p<0.01, ***p<0.001, n.s., not significant.

Figure S6. Siglec-F has no effect on bacterial clearance, Related to Figure 1. We used BALB/c mice as wild-type controls since *Siglecf*$^{-/-}$ mice are on the BALB/c background. (**A**) Kaplan-Meier curve for *Siglecf*$^{-/-}$ and BALB/c wild-type mice after i.p. injection with *E. coli 25922* (n=8). Log-rank (Mantel-Cox) test. (**B**) Cytokine production in blood measured at 16 h post-infection. (n=5). (**C**) Bacterial loads in spleen and liver after i.p. injection with *E. coli 25922* for 16 h. (n=4). Data are presented as the mean ± S.E.M from two independent experiments, Student's t-test, n.s., not significant.

Figure S7. Flow cytometric analysis of neutrophils isolated from mouse bone marrow, Related to Figure 2. Neutrophils were isolated from mouse femurs as described previously (Swamydas and Lionakis, 2013). The purity of the cells was analyzed by staining with CD11b and Gr-1 antibodies. As shown, neutrophils collected from the interface were >90% pure, and no difference in purity was observed between the two genotypes. Representative FACS profiles are shown. Experiments in this figure were reproduced three times.

Figure S8. Flow cytometric analysis of bacteria labeled with CFSE (**A**), ***E. coli 25922GFP*** (**B**) **, Related to Figure 2**. Bacteria were collected after overnight culture and washed twice in PBS. The pellet was suspended with 2 ml 10 µM CFSE (Sigma) in PBS, incubated at room temperature for 1 h, washed three times with PBS, and resuspended in PBS. The labeled bacteria were diluted, plated on agar plates and counted. For heat-killed bacteria, bacteria were incubated at 65°C for 20 min and stored at 4°C for later use.

Figure S9. Flow cytometric analysis of uptake and phagocytosis of bacteria in peritoneal macrophages, Related to Figure 2. Macrophages were collected from peritoneal washes and then incubated with *E. coli 25922 GFP* or CFSE-labeled live or heat-treated bacteria (MOI = 100) for 60 min in antibiotic-free medium, after which cells were washed to remove non-phagocytosed bacteria. Phagocytosed bacteria were measured by flow cytometry. Representative FACS profiles are shown. Experiments in this figure were reproduced three times.

Figure S10. Flow cytometric analysis of uptake and phagocytosis of bacteria in Trypan treated or untreated peritoneal macrophages after bacterial infection, Related to Figure 2. Macrophages were collected from peritoneal washes and then incubated with CFSE-labeled live or heat-treated bacteria (MOI = 100) for 30 min in antibiotic-free medium, after which cells were

washed to remove non-phagocytosed bacteria and then treated with trypan or untreated. Phagocytosed bacteria were measured by flow cytometry. Representative FACS profiles are shown. Experiments in this figure were reproduced three times.

Figure S11. Establishing stable cell lines expressing of Siglec-E mutants in Raw264.7 cells, Related to Figure 4. (**A**) Schematic map of mutation in Siglec-E. (**B**) Flow cytometric analysis of Siglec-E expression on Raw264.7 cells. As shown, same level of Siglec-E expressed on Raw264.7 cells. The bar graphs show the mean ± S.E.M MFI value from one representative experiment (n=3). Experiments in this figure were reproduced three times. Student's t-test, n.s., not significant. Iso con: isotype control.

Figure S12. Evaluation of Siglec expression in bacteria infected-THP-1 cells by real-time PCR using Siglec primer sets, Related to Figure 6. A, THP-1 cells were infected with indicated bacteria (MOI = 100) for 5 h, and the expression of Siglecs was analyzed by real-time PCR. The bar graphs show the mean ± S.E.M. Experiments in this figure were reproduced two times. **B**, THP-1 cells were transfected with vectors expressing shRNA for Siglec-9 or scramble, and the expression of siglec-9 was analyzed by real-time PCR. The bar graphs show the mean ± S.E.M. Experiments in this figure were reproduced two times. **C**, ROS production after bacterial infection. THP-1 cells were infected with *E. coli 25922* (MOI of 100:1) for 5 h at 37°C in vitro. ROS production was detected with H_2DCFDA. Data are presented as the mean ± S.E.M from two independent experiments, Student's t-test, ***p<0.001.

Figure S13. Siglec-E promoter and its putative AP-1 binding sites at 710 bp and 740 bp upstream of the translational start site (as +1) , Related to Figure 7. AP-1 consensus binding sites are underlined. Mutated nucleotides in Mut1 and Mut2 are shown in lowercase.

Figure S14. Evaluation of JNK and Syk expression in Raw264.7 cells, Related to Figure 7. Raw264.7 cells were treated with in JNK or Syk siRNA or control siRNA for 48 h, and the expression of JNK or Syk was analyzed by real-time PCR using primer sets for JNK (**A**) or Syk (**B**), respectively. The bar graphs show the mean ± S.E.M. Experiments in this figure were reproduced two times.

REFERENCES

SWAMYDAS, M. & LIONAKIS, M. S. 2013. Isolation, purification and labeling of mouse bone marrow neutrophils for functional studies and adoptive transfer experiments. *J Vis Exp*, e50586.

Transparent Methods

Reagents

Anti-Siglec-E-APC, anti-Siglec-F and anti-Gr-1(Ly-6G/Ly-6C) antibodies were purchased from BioLegend (San Diego, CA). Anti-mouse CD11b, CD4, CD8, and B220 antibodies were purchased from BD Bioscience (San Jose, CA). The following supplies were purchased from Santa Cruz Biotechnology (Santa Cruz, CA): anti-Syk, JNK, P-JNK, P38, P-P38, Erk, P-ERK, and β-actin; Streptavidin-horseradish peroxidase (HRP) and HRP-conjugated anti-mouse, anti-goat or anti-rabbit secondary antibodies; and JNK, Syk, and control siRNA. Lentiviral vectors expressing Siglec-E shRNA or Siglec-9 shRNA were from Thermo Scientific (Waltham, MA). Puromycin was purchased from Sigma. Blasticidin was obtained from InvivoGen (San Diego, CA). LPS (from E. coli 0111:B4) was from Sigma-Aldrich (St. Louis, MO). Biotinylated *Maackia amurensis* lectin II (MAL II) and biotinylated SNA (EBL) were purchased from Vector Laboratories (Burlingame, CA). RAW264.7 cells were obtained from ATCC (Manassas, VA) and cultured in Dulbecco's Modified Eagle's Medium (Thermo Fisher Scientific, Waltham, MA) supplemented with 10% heat-inactivated fetal bovine serum, 2 mM glutamine, 100 µg/ml penicillin and streptomycin. Syk inhibitor piceatannol, JNK inhibitor SP600125, and NF-kB inhibitor Bay11-7085 were purchased from Santa Cruz Biotechnology. Anti-Siglec-1 antibodies and Siglec-E Fc were obtained from R&D Systems (Minneapolis, MN) or prepared as previously reported (Chen et al., 2014).

Construction of plasmids

To generate a construct expressing mouse Siglec-E, cDNA for Siglec-E was amplified by RT-PCR and subcloned into expression vector pCDNA6 (Life Technologies, Carlsbad, CA). Siglec-

E mutants were made by using a QUIKCHANGE II XL SITE-DIRECTED MUTAGENES kit (Agilent Technologies, Santa Clara, CA) with the primers [M-1 (R126D): TTATACTTCTTTGACCTGGAGCGTGGA, TCCACGCTCCAGGTCAAAGAAGTATAA; M-2 (Y432F): GAAGAGATACATTTTGCGACCCTCAGC, GCTGAGGGTCGCAAAATGTATCTCTTC; M-3 (Y455F): ACTACCACGGAGTTCTCAGAGATAAAG, CTTTATCTCTGAGAACTCCGTGGTAGT; M4 (Y432F and Y455F)]. The shRNA targeted site in these expression vectors was further mutated with the primer (ttgagcctgtctccacagagctcagccaccctgtcggagatgatgatggggacctttg) without changing an amino acid. Primers used for generating constructs expressing wild-type and AP-1 binding site mutant Siglec-E promoter included: SE wild-type: cccgggAGCGTCAGTTGGGGAAGTGCCTCC; gagctCAGCATGTCCAGCTAAAACTGTCTC; SE Ap1mut1: TCCCCGACaCAaaCATTGACTGATCAGCTT, AAGCTGATCAGTCAATGttTGtGTCGGGGA; SE Ap1mut2: TGATCAGCTTCTTTATTGgCCAATCAGGGA, TCCCTGATTGGcCAATAAAGAAGCTGATCA. All constructs were verified by restriction enzyme digestion and DNA sequencing.

Cell culture and lentivirus infection

A GFP lentiviral vector expressing Siglec-E shRNA was transfected into Raw264.7 cells. Stable clones were obtained after selection with puromycin (2.5 μg/ml) for 3 weeks after infection. One clone, with knockdown efficiency confirmed by flow cytometry, was transfected with the expression vectors to make Raw264.7 stable cell lines overexpressing wild-type Siglec-E; mutants M-1, M-2, M-3 and M-4; or empty vector. Stable clones were obtained after selection with Blasticidin. Lentiviral vectors expressing Siglec-9 shRNA were transfected into THP-1 cells, and stable clones were obtained after selection with puromycin.

Experimental animal models

All mice used were 6-8 weeks of age. Age- and sex-matched wild-type littermates were used as controls for Siglec-E knockout mice. The Siglec-E knockout mouse generated with 129/Sv ES cells was backcrossed to C57BL/6. Siglec-E deficient mice have been described (Wu et al., 2016b, Chen et al., 2014), and the mice appeared healthy and did not display gross abnormalities. It is difficult to rule out the influence of 129-derived passenger gene mutations even after more than 10 backcross generations (Vanden Berghe et al., 2015) because of the efficiency of genetic recombination. Nevertheless, genetic recombination is highly unlikely in the region flanking the targeted allele (Lusis et al., 2007, Holmdahl and Malissen, 2012, Vanden Berghe et al., 2015). The *Casp11* gene contains a 129/Sv passenger mutation (Vanden Berghe et al., 2015, Broz et al., 2012). We previously typed *Casp11* and excluded *Casp11* mutation in Siglec-E knockout mice (Chen et al., 2014). Additionally, the expression and function of TLR4 and TLR2 were unaffected in Siglec-E knockout in our recent studies (Wu et al., 2016b). Furthermore, we confirmed Siglec-E knockout mice were backcrossed to C57BL/6 for 8 generations by genotyping single nucleotide polymorphisms (SNPs; MGI SNP database) located in the region flanking the Siglec-E targeted allele (the SNPs located 30 Mb upstream and 30 Mb downstream of Siglec-E were sequenced and confirmed) that distinguish the 129/Sv and C57BL/6 genomes (Figure S1). Moreover, Siglec-E knockout mice used were from more than 8 backcross generations, and wild-type littermates were used as controls in all the experiments. All animal procedures were approved by the Animal Care and Use Committee of University of Tennessee Health Science Center. Wild-type C57BL/6J, Siglec-F, MyD88, TLR2 and TLR4 knockout and FOPX3$^{IRES\text{-}GFP}$ mice were obtained from The Jackson Laboratory (Bar Harbor, ME).

Immunofluorescence microscopy

Spleen, liver or kidney was embedded in OCT compound and cryosectioned at 5 µm. Images were acquired with an EVOS FL Auto Imaging System (Thermo Fisher Scientific).

Bacterial culture

E. coli 25922 (ATCC 25922), *E. coli 25922GFP* (ATCC 25922GFP) (this clone derived from *E. coli 25922* contains a multicopy vector encoding GFPmut3), *L. monocytogenes* (ATCC 19115) and *S. aureus* (ATCC 25923) were obtained from ATCC and propagated according to the manufacturer's protocol. Strains were grown overnight in Luria-Bertani (LB) broth, Brain Heart Infusion Agar/Broth or LB nutrient broth. In the logarithmic phase of the growth, the suspension was centrifuged at 1000 x g for 15 min, the supernatant was discarded, and the bacteria were resuspended and diluted with sterile 1 x PBS.

In vivo bacterial infections and enumeration of bacterial burdens

For i.p. infection, female mice were infected with at a dose of 5 x 10^5 colony-forming units (c.f.u.) *E. coli 25922* or *E. coli 25922GFP*, 1 x 10^7 c.f.u. *DH5α*, 1 x 10^6 c.f.u. *S. aureus*, or 1 x 10^6 c.f.u. *L. monocytogenes* unless otherwise specified. For i.v. infection, female mice were infected at a dose of 1 x 10^5 c.f.u. *E. coli 25922GFP*. Tissues were collected 16 h post-infection and homogenized. Dilutions were plated on LB agar. Bacterial numbers are expressed as c.f.u g^{-1} tissue.

In vitro bacterial infections

Neutrophils were isolated from mouse femurs (Swamydas and Lionakis, 2013). Infections of neutrophils for an in vitro growth assay were at multiplicity of infection (MOI) of 100:1. Neutrophils were co-incubated with *E. coli 25922GFP* or CFSE-labeled live or heat-treated bacteria for 60 min in antibiotic-free medium. Next, cells were washed to remove non-phagocytosed bacteria. Phagocytosed bacteria were measured by flow cytometry. Gentamycin (100 μg ml^{-1}; Sigma-Aldrich) was added to the medium, and neutrophils were collected after 30-, 90-, 150-, 210-, and 270-min incubations. The cells were lysed with 0.2% Triton X-100, and c.f.u was measured.

ROS staining by flow cytometry

For detecting ROS levels, spleen cells from bacteria-infected mice or cultured cells were incubated with H_2DCFDA for 15 min (10 μM, Life Technologies). After incubation, the levels of fluorescence were measured by flow cytometry.

Analysis of immune cell infiltration in the peritoneum

Mice were infected with the indicated bacteria, and the cells were collected by peritoneal lavage in 6 ml PBS, washed twice with PBS and surface-stained for anti-CD11b and anti-Gr-1. Monocytes were characterized as $CD11b^{+}Gr\text{-}1^{-}$ cells, whereas $CD11b^{+}Gr\text{-}1^{+}$ cells were considered neutrophils.

Flow cytometry

Spleen cells from wild-type or Siglec-E knockout mice treated with PBS, *E. coli* 25922 or *E. coli* 25922GFP, *DH5α*, *L. monocytogenes*, *S. aureus* or cultured cells were washed with flow cytometry staining buffer (1 x PBS, 2% BSA), and then incubated for 1 h on ice with different

directly conjugated antibodies. The fluorescence intensity of cells was analyzed on LSRFortess Flow cytometer or Guava easyCyte^{TM} System (EMD Millipore, Merck KGaA, Darmstadt, Germany).

Real-time quantitative PCR

Total RNA was extracted with TRIzol (Invitrogen, Carlsbad, CA) according to the manufacturer's protocol and reverse transcribed with random primers and Superscript III (Life Technologies). The mRNA expression of mouse and human Siglecs, JNK and Syk was measured by real-time polymerase chain reaction. Samples were run in triplicate, and the relative expression was determined by normalizing expression of each target to the endogenous reference, hypoxanthine phosphoribosyltransferase (Hprt) transcripts. Real-time PCR primers used for mouse and human Siglecs were described previously (Wu et al., 2016b, Chen et al., 2014, Wu et al., 2016a). Real-time PCR primers used for mouse JNK were ATGGCTGTCGATATTCAACCAG, CCTCTTGGGCATACCCCAC and for Syk were CTACCTGCTACGCCAGAGC, GCCATTAAGTTCCCTCTCGATG.

Immunoblotting

RAW264.7 cells or neutrophils lysates were prepared in lysis buffer (20 mM Tris-HCl, 150 mM NaCl, 1 % Triton X-100, pH 7.6, including protease inhibitors, 1 μg ml^{-1} leupeptin, 1 μg ml^{-1} aprotinin and 1 mM phenylmethylsulfonyl fluoride), sonicated, centrifuged at 13,000 rpm for 5 min and then applied for Western blot analysis. The concentration of running gel was 10%. After blocking, the blots were incubated with primary antibody (1:1,000 dilution). After incubation with the second antibody (HRP-conjugated goat anti-rat IgG, rabbit anti-goat IgG, or

goat anti-mouse IgG) (1:5,000 dilution), the signal was detected with an ECL kit (Santa Cruz, CA).

Measurement of inflammatory cytokines

Blood samples were obtained at indicated time points, and cytokines in the serum were determined using a mouse cytokine bead array designed for inflammatory cytokines (BD Biosciences, 552364).

Statistical analysis

The differences in cytokine concentrations and bacterial clearance were analyzed by two-tailed t-tests in single pairwise comparisons calculated with Excel (Microsoft). Data are shown as the mean ± SEM. The differences in survival rates were analyzed by Kaplan-Meier plots, and statistical significance was determined using a log-rank (Mantel-Cox) test (GraphPad Software, San Diego, CA). *P<0.05, **P<0.01, ***P<0.001, n.s., not significant.

REFERENCES

BROZ, P., RUBY, T., BELHOCINE, K., BOULEY, D. M., KAYAGAKI, N., DIXIT, V. M. & MONACK, D. M. 2012. Caspase-11 increases susceptibility to Salmonella infection in the absence of caspase-1. *Nature,* 490**,** 288-91.

CHEN, G. Y., BROWN, N. K., WU, W., KHEDRI, Z., YU, H., CHEN, X., VAN DE VLEKKERT, D., D'AZZO, A., ZHENG, P. & LIU, Y. 2014. Broad and direct interaction between TLR and Siglec families of pattern recognition receptors and its regulation by Neu1. *Elife,* 3**,** e04066.

HOLMDAHL, R. & MALISSEN, B. 2012. The need for littermate controls. *Eur J Immunol,* 42**,** 45-7.

LUSIS, A. J., YU, J. & WANG, S. S. 2007. The problem of passenger genes in transgenic mice. *Arterioscler Thromb Vasc Biol,* 27**,** 2100-3.

SWAMYDAS, M. & LIONAKIS, M. S. 2013. Isolation, purification and labeling of mouse bone marrow neutrophils for functional studies and adoptive transfer experiments. *J Vis Exp***,** e50586.

VANDEN BERGHE, T., HULPIAU, P., MARTENS, L., VANDENBROUCKE, R. E., VAN WONTERGHEM, E., PERRY, S. W., BRUGGEMAN, I., DIVERT, T., CHOI, S. M., VUYLSTEKE, M., SHESTOPALOV, V. I., LIBERT, C. & VANDENABEELE, P. 2015. Passenger Mutations Confound Interpretation of All Genetically Modified Congenic Mice. *Immunity,* 43**,** 200-9.

WU, Y., LAN, C., REN, D. & CHEN, G. Y. 2016a. Induction of Siglec-1 by Endotoxin Tolerance Suppresses the Innate Immune Response by Promoting TGF-beta1 Production. *J Biol Chem,* 291**,** 12370-82.

WU, Y., REN, D. & CHEN, G. Y. 2016b. Siglec-E Negatively Regulates the Activation of TLR4 by Controlling Its Endocytosis. *J Immunol*.
